# Landscape Genomics and Evolutionary History of *Megamelus scutellaris*, a Biocontrol Agent of the Invasive Water Hyacinth (*Pontederia crassipes*)

**DOI:** 10.1111/eva.70208

**Published:** 2026-02-18

**Authors:** Nicolas A. Salinas, Daniel Poveda‐Martínez, Marcela S. Rodriguero, Melissa C. Smith, María E. Brentassi, Alejandro J. Sosa

**Affiliations:** ^1^ Fundación Para el Estudio de Especies Invasivas (FuEDEI), Hurlingham Buenos Aires Argentina; ^2^ Consejo Nacional de Investigaciones Científicas y Técnicas (CONICET), CABA Buenos Aires Argentina; ^3^ Centre de Biologie Pour la Gestion Des Populations (CBGP‐IRD) Montpellier France; ^4^ Instituto de Ecología, Genética y Evolución de Buenos Aires (IEGEBA) Buenos Aires Argentina; ^5^ Departamento de Ecología, Genética y Evolución FCEN‐UBA Buenos Aires Argentina; ^6^ USDA‐ARS Invasive Plant Research Laboratory Davie Florida USA; ^7^ División Entomología Facultad de Ciencias Naturales y Museo, Universidad Nacional de La Plata Buenos Aires Argentina; ^8^ Comisión de Investigaciones Científicas de la Provincia de Buenos Aires Buenos Aires Argentina

**Keywords:** demographic history, invasion biology, phylogeography, planthoppers, population genomics

## Abstract

Understanding the evolutionary history of biological control agents in their native ranges is crucial for improving their selection, establishment, and performance across environmentally diverse regions. Phytophagous insects that specialize on aquatic plants offer particularly valuable models, as their evolutionary trajectories may be shaped by a combination of climatic variation, host plant availability, and the fragmented nature of aquatic habitats. *Megamelus scutellaris* is a monophagous planthopper native to South America that has been introduced into the United States and South Africa as part of biological control programs targeting the highly invasive aquatic plant, *Pontederia crassipes*. In this work, we combined nuclear SNP and mitochondrial sequence data to investigate the genetic structure, demographic history, and environmental drivers of population divergence in *M. scutellaris* across its native range in Argentina and Paraguay. We identified three main genetic lineages broadly associated with major river basins and ecoregions. Demographic modeling supported an early divergence, likely linked to Pleistocene climatic shifts and hydrological changes, followed by a more recent split dated to the early Holocene. Contemporary gene flow was asymmetric and varied in magnitude among lineages, reflecting differences in connectivity and environmental conditions. Lastly, landscape genomic analyzes revealed a strong association between genetic differentiation and climatic variation, supporting models of isolation by environment and resistance. These findings highlight the role of evolutionary and ecological processes in shaping the genetic landscape of *M. scutellaris* and provide key insights for selecting source populations better suited to different environments in introduced regions.

## 1 Introduction

The distribution of insect species is shaped by climatic, geological, and environmental factors that vary across space and time. For monophagous herbivorous insects in particular, host plant distribution is a primary determinant of habitat availability and dispersal potential. These ecological and evolutionary forces, together with species‐specific traits, influence patterns of genetic variation, population structure, and local adaptation (Funk et al. 2006; Forbes et al. 2017; Ortego et al. 2017; Vidal et al. 2019). Understanding these dynamics is crucial to the biological control (BC) of plants, where the selection of insects as natural enemies benefits from assessing their genetic diversity, ecological flexibility, and evolutionary potential to establish and persist in new environments.

Classical biological control with herbivorous insects is a widely used approach to reduce populations of invasive exotic plant species (Van Driesche et al. 2010). This scientific discipline focuses on selecting host‐specific specialist herbivores from the native range of a target weed for their release in regions where the plant has become invasive (Culliney 2005), providing an environmentally sustainable effective alternative to chemical and mechanical control methods (Culliney 2005). However, the implementation of BC programs faces some challenges. These include ensuring the taxonomic identity of the introduced species and its specificity for its host; potential non‐target effects on native plants or other organisms; and the long‐term success of the control agents in the new environment (Hill and Coetzee 2017; Hinz et al. 2020).

Until recently, BC programs primarily relied on morphological and ecological assessments to tackle these issues. Advances in insect genomics now offer deeper insights into the biology, behavior, and ecology of natural enemies, enabling a more precise identification, selection, and monitoring of agents that are both effective in controlling invasive plants and sufficiently host‐specific (Gaskin et al. 2011; Leung et al. 2020; Sethuraman et al. 2020; Hinz et al. 2024). The integration of genomic technologies may help to understand the influence of ecological and evolutionary processes, and thus contribute to optimizing BC programs (Gaskin et al. 2011; Müller‐Schärer et al. 2020), ensuring more sustainable and successful invasive plant management.

Water hyacinth (*Pontederia* (syn. *Eichhornia*) *crassipes* Mart. (Pontederiaceae)), a floating, perennial plant, is globally recognized as one of the most invasive aquatic plant species (Lowe et al. 2000; Roy et al. 2023). Native to South America, it has been spread intercontinentally since the late 1800s as an ornamental plant (Hill and Coetzee 2008), and now extends from 51° N (Russia) to 40° S (New Zealand) (Center et al. 2002; Philippov et al. 2024). Amongst other negative socio‐economic and ecological impacts, water hyacinth invasions decrease water quality and biodiversity, alter habitat for pathogen vectors, and impede ship navigation and water cycling at hydroelectric and water treatment facilities (Schmitz et al. 1993).

To control the plant, *Megamelus scutellaris* Berg (Hemiptera: Delphacidae), a planthopper that feeds and reproduces exclusively on it (Sosa et al. 2004, 2007), was introduced as a BC agent in the United States and South Africa in 2010 and 2013, respectively (Tipping et al. 2014; Coetzee et al. 2022). This planthopper is native to the wetlands across South America, with records spanning from the Amazon River basin to the Río de la Plata basin, including Argentina, Uruguay, northern Peru, and southern Brazil (Sosa et al. 2004).

The first specimens of *M. scutellaris* exported to be used for BC were collected in 2006 from Otamendi, in the Paraná River Delta of Buenos Aires province, a temperate region in Argentina (Foley et al. 2016). These insects were reared in the quarantine facilities of the USDA‐ARS Invasive Plant Research Laboratory (IPRL) in Ft. Lauderdale, Florida (US), and then field released in 2010 (Tipping et al. 2014). Insects from this rearing effort were later shipped to California (US) in 2011 (Moran et al. 2016) and the Eastern Cape (South Africa) in 2013, from where they were further distributed across different sites in South Africa invaded by water hyacinth (Miller et al. 2023). While *M. scutellaris* successfully established in most locations, its densities remained consistently lower in southern, subtropical sites in Florida compared to more temperate sites in the northern part of the state. This led to a second introduction, sourcing a new population from Arroyos y Esteros, in the Department of Cordillera, Paraguay in 2013, with the expectation that it would better tolerate Florida's high temperatures (Foley et al. 2016). This population successfully established in Florida (Goode et al. 2021). Subsequent laboratory experiments comparing these two colonies, sourced from Argentina and Paraguay, revealed differences in the insects' performance across seasonal temperature changes (Foley et al. 2016). These phenotypic differences between introduced populations, along with the varying effectiveness of *M. scutellaris* as a BC agent in different environments, strongly suggest underlying genetic differentiation among native populations.

**FIGURE 1:**
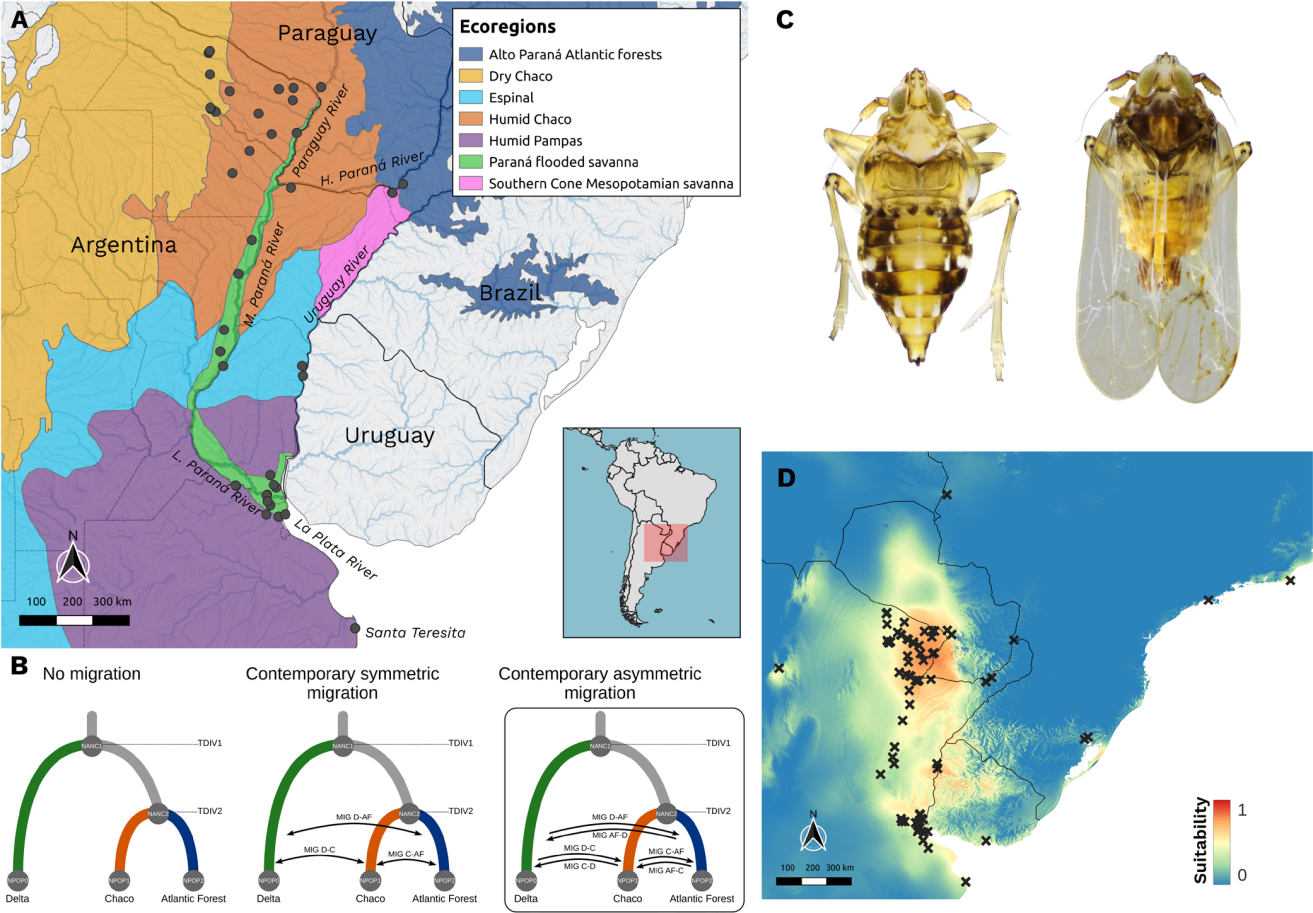
Study area and demographic modeling. (A) Map of the study area including main rivers (in italics) and ecoregions according to Dinerstein et al. (2017). Gray dots represent sites where *Megamelus scutellaris* samples were collected (Table S1). Tags in italics represent main rivers of the Río de la Plata basin and the sampling locality of Santa Teresita. (B) Post divergence migration models tested using coalescent‐based simulations in fastsimcoal2. Parameters include ancestral (N_ANC1_, N_ANC2_) and contemporary (N_POP0_, N_POP1_, N_POP2_) effective population sizes, migration rates (MIG_D‐AF_, MIG_D‐C_, MIG_C‐AF_, MIG_AF‐D_, MIG_C‐D_, MIG_AF‐C_) and timing of population divergence (T_DIV1_ and T_DIV2_). The model outlined in the box corresponds to the best‐supported scenario, based on AIC. (C) Map of habitat suitability for *M. scutellaris* based on bioclimatic variables. Warmer colors indicate higher habitat suitability. Crosses indicate occurrence records used for modeling. (D) Brachypterous female (left) and macropterous male (right) of *M. scutellaris* .

Across its native range, population structure in *M. scutellaris* may be shaped by environmental and geographic factors such as climate, host plant availability, and the distribution of aquatic habitats. For instance, separation across distinct river systems may restrict dispersal and promote genetic differentiation, as observed in other river‐associated insects (McCulloch et al. 2022). However, geographic isolation alone does not necessarily result in genetic differentiation. This depends largely on dispersal ability, with more mobile species exhibiting greater gene flow, which can counteract genetic drift and maintain genetic connectivity even across spatially separated habitats (Phillipsen et al. 2015). Like other delphacids, adult *M. scutellaris* exhibit wing dimorphism: some individuals are brachypterous (possessing reduced, non‐functional wings) while others are macropterous (with fully developed wings suited for dispersal, Figure 1C) (Sosa et al. 2004). A thorough genetic study of *M. scutellaris* is therefore crucial to uncover the extent of this divergence and to identify genetic groups within its native range. Such information is essential for optimizing BC strategies, as understanding the genetic structure and historical divergence of *M. scutellaris* can guide the selection of source populations that are more likely to establish and perform effectively under the environmental conditions of invaded regions, ultimately improving the success of water hyacinth management programs. Moreover, clarifying the evolutionary and demographic context of native populations provides a foundation for future studies on potential local adaptation within the native range, as well as on the ecological and genetic processes shaping introduced populations.

In this study, we combined nuclear genome‐wide SNP data (nDNA) with mitochondrial DNA (mtDNA) to evaluate the genetic variation of *M. scutellaris* in its native range. First, we examined the population structure and phylogeographic patterns of this planthopper across major river basins in Argentina and southern Paraguay. Next, we assessed the divergence and connectivity of the inferred genetic groups through a coalescent‐based simulation framework, enabling a more precise understanding of their demographic history. Finally, we integrated a landscape genomics approach with ecological niche modeling to identify potential environmental factors shaping the observed genetic structure. We hypothesize that *M. scutellaris* populations will exhibit genetic differentiation driven by geographic distance, directional river flow, and/or environmental heterogeneity. In particular, these factors may restrict effective gene flow and promote spatial structure in this planthopper, despite the continuous aquatic landscape that could otherwise facilitate dispersal and connectivity across the river system. Taken together, our results reveal how historical and environmental factors have shaped genetic structure in *M. scutellaris* but also establish a valuable foundation for improving its efficacy as a biological control agent.

## 2 Methods

### 2.1 Study Area, Sample Collection and DNA Extraction

Field surveys were conducted in Argentina and Paraguay from December to May between 2021 and 2023, across the known native range of *M. scutellaris* . Our study encompassed sites within the Río de la Plata river basin, which includes the Paraná, Paraguay, Uruguay, and La Plata rivers and their tributaries (Figure 1A; Table S1). The Paraná river can be divided into four stretches: the Upper Paraná, which runs through Brazil; the High Paraná, flowing from the Itaipú Reservoir to the confluence with the Paraguay River; the Middle Paraná, stretching southwards to the city of Diamante; and the Lower Paraná, which flows from Diamante and ends in a vast Delta of islands before joining the Uruguay River to form La Plata River (Bonetto et al. 1986; Devercelli and de Domitrovic 2014). Sampling sites located across seven Argentine provinces (Buenos Aires, Entre Ríos, Corrientes, Misiones, Chaco, Formosa and Santa Fe) and one department from Paraguay (Presidente Hayes) and occur in the following ecoregions: Humid Pampas, Espinal, Humid Chaco, Dry Chaco, Alto Paraná Atlantic forests, Southern Cone Mesopotamian savanna and the Paraná flooded savanna (Dinerstein et al. 2017) (Figure 1A). Sampling included the areas from which the biological control populations in the U.S. and South Africa were originally sourced. Although no water hyacinth was found on the original site in Arroyos y Esteros (Paraguay), we sampled *M. scutellaris* approximately 50 km away from that locality.

Insects were collected from *P. crassipes* mats located in the aforementioned rivers and in water bodies with public access such as streams, lagoons, marshes, and ditches. Adults were collected directly from the host plant using manual aspirators. When possible, individuals were collected from plants located at about 5–10 m apart, up to a total of 4–5 points per site to avoid sibling sampling. Samples were immediately placed in absolute ethanol and stored at −20°C for later taxonomic identifications (Salinas et al. 2025) and subsequent DNA extraction.

Total genomic DNA was extracted from whole *M. scutellaris* bodies of adults of both sexes and winged forms (brachypters and macropters) using the QIAGEN DNeasy Blood & Tissue Kit (QIAGEN, Germany) according to the manufacturer's instructions. After the lysis step, 2 μL of RNAse A (Promega, USA) were added and samples were incubated at 37°C for 30 min. DNA concentration and quality was quantified using a DS‐11 Spectrophotometer/Fluorometer (Denovix, USA) and visualized in 1% agarose gels stained with GelRed (Biotium, USA).

### 2.2 Genomic Libraries Preparation, Illumina Sequencing and *de Novo* Reference Assembly

Total genomic DNA was converted into nextRAD genotyping‐by‐sequencing libraries (SNPsaurus LLC) as in Russello et al. (2015). Genomic DNA was first fragmented with Nextera Flex reagent (Illumina Inc), which also ligates short adapter sequences to the ends of the fragments. The Nextera reaction was scaled for fragmenting 20 ng of genomic DNA, although 30 ng of genomic DNA was used for input to compensate for the amount of degraded DNA in the samples and to increase fragment sizes. Fragmented DNA was then amplified for 27 cycles at 74°C, with one of the primers matching the adapter and extending 10 nucleotides into the genomic DNA with the selective sequence GTGTAGAGCC. Thus, only fragments starting with a sequence that can be hybridized by the selective sequence of the primer will be efficiently amplified. The nextRAD libraries were sequenced on a Novaseq 6000 with one SP lane of single‐end 122 bp reads (University of Oregon sequencing facilities, USA).

Raw sequence data were deposited in the National Center for Biotechnology Information (NCBI) (BioProject accession number: PRJNA1222173). Information for samples used in genomic analyzes, including collection dates and coordinates, are shown in Table S1.

A *de novo* reference was created by collecting 10 million reads in total, evenly from the samples (total of 190 samples including *M. scutellaris* and other *Megamelus* species), and excluding reads that had counts fewer than six or more than 600. The remaining loci were then aligned to each other to identify allelic loci and collapse allelic haplotypes to a single representative. This reference draft genome is available in a Figshare repository.

### 2.3 Genomic Data Processing

Raw reads from nextRAD sequencing were first pruned to remove Nextera adaptors using Trimmomatic v.0.39 (Bolger et al. 2014). Read quality and length were assessed for each sample with FastQC (Andrews 2010) and outputs compiled and summarized on MultiQC v1.12 (Ewels et al. 2016). Based on these reports, reads were processed with ipyrad 0.9.92 (Eaton and Overcast 2020), allowing a maximum read length of 120 bp, and a Phred quality score > 33. Filtered reads were mapped against the previously generated *de novo* reference. On subsequent ipyrad assembly steps, 20% of SNPs per locus were allowed, polymorphic sites occurring across a maximum of 50% of samples, and minimum number of samples per locus representing 50% of the total. The resulting Variant Call Format (VCF) file was then filtered using VCFtools v.0.1.16 (Danecek et al. 2011) based on the following criteria: minimum and maximum number of alleles (2), maximum missing data per site (< 20%) and read depth per site (min = 10X—max = 70X), and minor allele frequency (MAF) of 0.03. Additionally, monomorphic SNPs were excluded for further analyzes. To prune SNPs in linkage disequilibrium we used PLINK v1.9 (Purcell et al. 2007) with recommended parameters: window size (50), window shift (5) and VIF threshold (2). Lastly, SNPs under selection were identified using Bayescan v.2.1 (Foll and Gaggiotti 2008) and excluded from the dataset.

### 2.4 Mitochondrial PCR Amplification and Sequencing

A fragment of 658 bp of the mitochondrial gene, cytochrome c oxidase subunit I (*COI*) barcode, gene was amplified using primers LepF2_t1 and LepR1 (Hebert et al. 2004), extensively used for Hemiptera barcoding (Park et al. 2011). Under the reaction conditions used for amplification, this primer pair also amplified a bacterial sequence belonging to the genus *Rickettsia*, revealing the presence of this bacterium in many of the samples. Thus, when the insect fragment amplification with primer pair LepF2_t1/LepR1 failed, the universal primer pair LCO/HCO (Folmer et al. 1994) was used instead. For both primer pairs, PCR amplification was carried out following (Salinas et al. 2025). PCR products were checked on agarose gels and purified by adding 0.5 μL (10 u) of Exonuclease I (Exo I) (Thermo Scientific, USA) and 1 μL (1 U) of Shrimp Alkaline Phosphatase (SAP) (Thermo Scientific, USA). Samples were incubated at 37°C for 15 min and the reaction was stopped by heating the mixture at 85°C for 15 min. Sanger sequencing of the samples was performed at Macrogen (South Korea) with the same primers used for PCR amplification. Posterior quality check and primer trimming were performed with CodonCode Aligner V. 10.0.2 (CodonCode Corporation, USA). Alignment of sequences was performed using the MUSCLE algorithm as implemented in MEGA v.11 (Tamura et al. 2021), with default settings.

### 2.5 Population Structure and Diversity

nDNA data was used to estimate genetic diversity for each sampling locality by means of expected heterozygosity (H_E_), observed heterozygosity (H_O_), inbreeding coefficient (F_IS_) and number of private alleles (P_A_). H_E_, H_O_ and F_IS_ were calculated with the populations program from STACKS (Catchen et al. 2013), while P_A_ were computed with the package *hierfstat* (Goudet 2005) implemented in R 4.4.2 (R Core Team 2024) and run in RStudio (Posit Team 2023). Sampling sites at close proximity of each other were combined into a single sampling locality (Table S2). Localities with fewer than three individuals were excluded from these analyzes, but were included in the assessment of the spatial structure of genetic variation (PCA and sNMF).

Population genetic structure was inferred using a Principal Component Analysis (PCA) based on the nDNA dataset, conducted with the R package *hierfstat* (Goudet 2005). Clustering analyzes were performed using the sparse non‐negative matrix factorization approach (sNMF), as implemented in the *LEA* R package (Frichot and François 2015), in order to assign samples to genetic clusters through the estimation of individual admixture coefficients. Such coefficients were calculated for K values ranging from 1 to 10, with 100 repetitions for each value of *K* and 9999 iterations. An optimal *K* value was determined using the cross‐entropy criterion based on the prediction of masked genotypes to evaluate the error of ancestry estimation.

**FIGURE 2:**
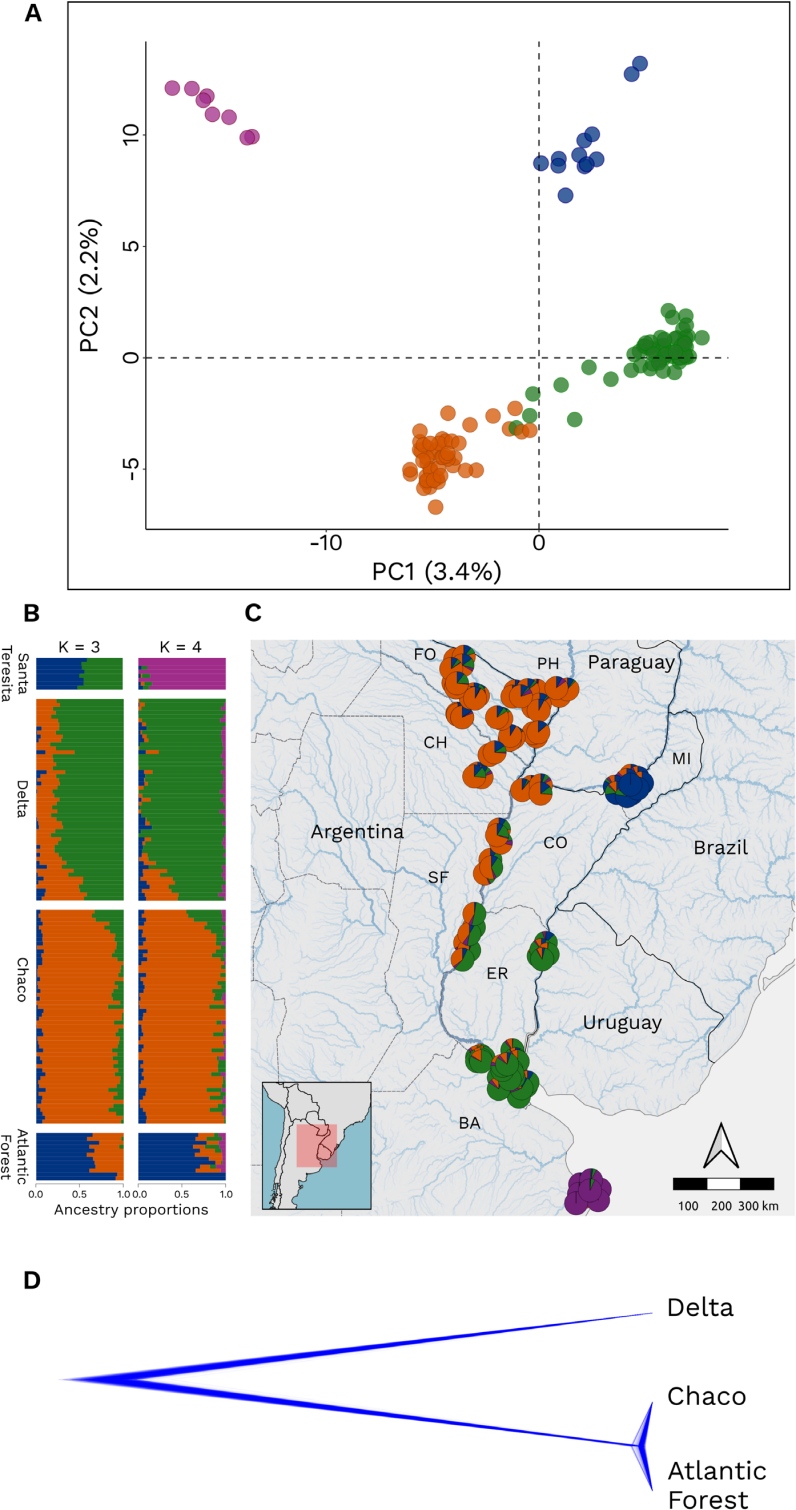
Population genetic structure and phylogenetic relationships of *M. scutellaris* based on nDNA data. (A) PCA showing four genetic groups. Each dot represents an individual colored by genetic groups based on sNMF. (B) Admixture proportions estimated with sNMF for *K* = 3 (left) and *K* = 4 (right). Each vertical bar corresponds to an individual. (C) Geographical distribution of sNMF ancestry coefficients (*K* = 4), displayed as pie charts at each sampling locality. (D) Coalescent‐based tree inferred with SNAPP, showing phylogenetic relationships among the three main genetic groups.

As a measure of genetic differentiation among the main genetic groups identified by sNMF and PCA analyzes (Figure 2A–C), fixation indexes (pairwise F_ST_) were calculated from nDNA data using the Weir and Cockerham (1984) method implemented in the *StAMPP* (Pembleton et al. 2013) R package, with 9999 bootstrap replicates. Pairwise *F*_ST_ values were calculated among groups using 12 individuals per genetic cluster, selected based on the lowest levels of admixture. Since the population from Santa Teresita, which was identified as a separate group by both PCA and sNMF, is believed to be a human‐mediated introduction, it was excluded from this and all downstream analyzes. To access mtDNA population structure, a minimum spanning network was built using PopART (Leigh and Bryant 2015).

### 2.6 Phylogenetic Analysis

The phylogenetic relationships among the main genetic groups identified by sNMF and PCA analyzes based on nDNA data were inferred using the coalescent‐based method SNAPP (Bryant et al. 2012) with default model parameters and U and V equal to one and running the analysis for 5,000,000 MCMC generations, sampling every 500 generations. From each of the three genetic clusters (Delta, Chaco, and Atlantic Forest; see Results section), we selected 12 individuals with the lowest degree of admixture to reduce computational burden. For the Delta and Chaco groups, individuals with a *q*‐value > 0.75 were chosen. For the Atlantic Forest group, however, the threshold was adjusted to *q*‐value > 0.55 due to the higher overall levels of admixture observed in this cluster. As this analysis implements the coalescent model, it does not require rooting to polarize the topology, allowing us to evaluate relationships among lineages without outgroups.

Additionally, phylogenetic relationships and divergence times were estimated from the mtDNA dataset using BEAST2 (Bouckaert et al. 2014), with a strict clock model, the Yule process as a prior tree and a substitution rate of 0.017 substitutions per site per million years (Papadopoulou et al. 2010). Sequences belonging to *Megamelus bellicus* Remes Lenicov & Sosa, 2007 (PP986913, PP986916), *Megamelus iphigeniae* Muir, 1926 (PP986920), *Megamelus maculipes* (Berg) (PP986927), *Megamelus delticus* Remes Lenicov & Mariani, 2025 (PP986941) and to the closely related *Conomelus anceps* (Germar) (HEMFI926‐15) and *Pissonotus paraguayensis* Bartlett (OR523788.1) were used as outgroups.

### 2.7 Demographic Inference

A coalescent simulation‐based approach implemented in *fastsimcoal2* v2.6 (*fsc2*) (Excoffier et al. 2013) was employed using the nDNA dataset to estimate the divergence times among the three main genetic groups identified by sNMF and PCA analysis, under three alternative scenarios of contemporary gene flow. For this analysis, we used the same 12 individuals with the lowest degree of admixture as in the SNAPP analysis. We tested three models with different scenarios of post‐divergence gene flow considering the topology obtained from SNAPP (Figure 1B): Model A describes a scenario with no gene flow among genetic groups (Null Model); model B represents a scenario with symmetric gene flow rates between all pairs of genetic groups, and model C includes a scenario with asymmetric gene flow among genetic groups.

The site frequency spectrum (SFS) was used to estimate the composite likelihood of the observed data given a specified model (Excoffier et al. 2013) with *fsc2*. Population size projection for the construction of the SFS from the final VCF file was performed using a custom script by Isaac Overcast (https://github.com/isaacovercast/easySFS) implemented in Python 3.11.4. A multiSFS for all populations was generated, selecting one SNP per locus to minimize the impact of linkage disequilibrium (Noguerales and Ortego 2022). Because invariable sites were not considered when generating the SFS, the contemporary effective population size for one of the three demes (N_POP1_ = Chaco, see Figure 1B) was fixed to enable the estimation of other parameters in *fsc2* (Excoffier et al. 2013). N_POP1_ was calculated according to the equation N_E_ = π/4 μ. Nucleotide diversity (π = 0.0047) was estimated using variant and invariant sites with DNAsp v12.03 (Rozas et al. 2017), and the empirical mutation rate (μ) of 2.8 × 10^−9^ estimated from *Drosophila melanogaster* (Keightley et al. 2014).

Models were run using 50 independent replicates, 40 cycles of the Brent algorithm, and 100,000 simulations for the calculation of the composite likelihood. The best demographic model was identified using Akaike's information criterion (AIC). AIC values were rescaled in terms of AIC differences (ΔAIC) according to the formula: ΔAIC*i* = AIC*i* − AIC*min* to compare models, where AIC*i* is the AIC value for model *i*, AIC*min* is the lowest AIC value among all compared models. ΔAIC*i* indicates how far model *i* is from the best model (the one with the lowest AIC). The model with a ΔAIC value of 0 and the highest AIC weight (ω*i*) was considered as the best. Finally, a parametric bootstrapping approach was used to construct 95% confidence intervals for the estimated parameters running 100 bootstrap replicates using initial values from the best model.

### 2.8 Landscape Genomics Analyzes

A landscape genomics approach was used to investigate potential factors that might account for the observed patterns of genetic differentiation in *M. scutellaris* . As a measure of genetic differentiation, pairwise F_ST_ between sampling localities was calculated from nDNA data using the same method as above (see Population structure and diversity section). Pairwise F_ST_ was calculated from the same 30 localities used for genetic diversity analyzes. We evaluated several plausible scenarios of population connectivity based on contemporary spatial and ecological data.

#### 2.8.1 Isolation by Environment

Climatic differences between localities were assessed to evaluate an isolation‐by‐environment scenario (IBE_CLI_). To this end, 19 bioclimatic variables were retrieved from the CHELSA v.1.2 database (https://chelsa‐climate.org/bioclim/) at 30 arc‐sec (~1 km) of resolution. However, four of them (Bio 8, 9, 18 and 19) were discarded for having artificial breaks (Oliveira et al. 2020). Environmental data was extracted from the remaining variables (see Table S3 for variable description) for each of the 30 sampling localities, as well as from 500 random points covering our study area to avoid potential biases resulting from only considering conditions at focal sites. Due to collinearity among the variables, we performed a PCA using the *ade4* R package (Chessel et al. 2004) and summarized the environmental variation in the three first axes, which explained 49.2%, 25.3% and 13.5% of the total variation (Figure S1), respectively. Finally, an environmental dissimilarity matrix was obtained by calculating the Euclidean distances for PC scores between pairs of sampling sites (Ortego et al. 2021).

#### 2.8.2 Isolation by Habitat Resistance

Different environmental layers were employed to build isolation‐by‐resistance (IBR) scenarios to assess the effect of landscape heterogeneity on the connectivity of *M. scutellaris* populations. First, we evaluated the role of climatic variation. To this end, an ecological niche model (ENM) for *M. scutellaris* was built based on the same bioclimatic variables used for IBE_CLI_ analyzes from the CHELSA v.1.2 database, using MaxEnt v.3.4.1 (Phillips et al. 2006) and the R package *kuenm* (Cobos et al. 2019) for model parameters selection and optimization. The model was built using the feature class combination ‘*no.t.h*,’ which excludes threshold and hinge features while incorporating linear, quadratic, and product features. Model performance was evaluated independently based on statistical significance (Partial ROC), omission rates (OR), and the Akaike information criterion corrected for small sample sizes (AICc). The partial ROC (Receiver Operating Characteristic) area under the curve (AUC) was used to assess discriminatory power relative to random expectations. For variable selection, variables that were highly correlated (*R* > 0.9) according to the variance inflation factor (VIF) criterion were excluded for model building, using the vifstep function from the *usdm* R package (Naimi et al. 2014). The final dataset used in the model was composed of five CHELSA variables: Bio 3, 5, 7, 13 and 14. Models performed better than random predictions (ROC AUC value = 0.918) (Figure 1D). Secondly, we assessed the influence of freshwater availability on population connectivity. Freshwater occurrence data was obtained from the Joint Research Centre's Global Surface Water Dataset (Pekel et al. 2016). This layer shows the frequency with which freshwater was present on the surface from March 1984 to December 2021. Lastly, we constructed an ENM for the host plant, *P. crassipes* , based on the CHELSA bioclimatic variables and freshwater occurrence data, using the previously mentioned approach for variable selection and model building (Figure S2). The final dataset used in the model was composed of six variables (Bio 2, 3, 5, 13, 15 and freshwater occurrence). Models performed better than random predictions (ROC AUC value = 0.850).

Occurrence data for both *M. scutellaris* and the host species were obtained from field surveys made from 1995 to 2023. For *P. crassipes* , our field data was complemented with distribution information available at GBIF (www.gbif.org, accessed September 27th, 2024), after curation of the records. Redundant occurrences (e.g., points located less than 2 km apart) were excluded using the R package *spThin* (Aiello‐Lammens et al. 2015). After thinning occurrence data, 77 records for *M. scutellaris* and 218 for *P. crassipes* were used to conduct ENMs (available in Figshare repository).

Circuit theory was used to model gene flow across a spatially variable landscape and assess the influence of different IBR scenarios on the observed genetic differentiation patterns. Resistance distances for all pairs of populations were calculated implementing an eight‐neighbor cell connection scheme in Circuitscape v.5 (Anantharaman et al. 2020), using the following raster layers as inputs: freshwater occurrence (IBR_WATER_), predicted distribution of the host plant (IBR_HOST_), and the aforementioned niche suitability map for *M. scutellaris* (IBR_ENM_). Pixel values of the rasters were assigned as conductance values in all cases. We also calculated resistance distances based on a “flat landscape” where all cells have an equal resistance value (= 1) representing a null model of isolation by resistance, which is equivalent to a model of isolation‐by‐distance (IBD) (Noguerales et al. 2016).

### 2.9 Statistical Analysis

Relationships between explanatory distance matrices based on landscape heterogeneity (IBD, IBE_CLIM_, IBR_WATER_, IBR_HOST_, IBR_ENM_) and the genetic differentiation among *M. scutellaris* localities (F_ST_) were explored using univariate analyzes and a multiple matrix regressions with randomization approach using the function MMRR (Wang 2013) as implemented in R (R Core Team 2024). Before building the models, all matrices were standardized to have a mean of zero and a standard deviation of one, ensuring comparability across different datasets. An initial full model was constructed considering all significant explanatory terms identified previously in univariate analyzes. Then, a final best‐fit model was selected using a backward‐stepwise procedure by progressively removing non‐significant variables until all retained terms within the model were significant (Wang 2013; e.g., Noguerales et al. 2016). The result was the minimal most adequate model for explaining the variability in the response variable, where only the significant explanatory terms were retained. Once again, the population from Santa Teresita was excluded from both genetic and explanatory matrices.

## 3 Results

### 3.1 Genomic Data

An average number of 2.6 million single‐end reads per individual was retained after quality control for 125 individuals. Details on per‐sample coverage, retained reads after filtering, reference‐mapped reads, and assembled loci are provided in Table S4. Reference‐based assembly and variant calling yielded 91,073 SNPs. The filtered dataset used for all downstream analyzes resulted in a matrix of 9198 biallelic unlinked neutral SNPs with a 32X average mean depth per individual. The numbers of loci removed in each filtering step are detailed in Table S5 while final datasets used in all analyzes are available in a Figshare repository.

### 3.2 Population Structure and Genetic Diversity

Principal component analysis revealed that samples were distributed among four clusters (Figure 2A). Individuals located in the Dry Chaco and Humid Chaco ecoregions and associated with the Paraguay River and its alluvial plain (hereafter, “Chaco” group) clustered together, while individuals located in the Lower Paraná Delta and Lower Uruguay River, in the ecoregions of the Humid Pampas and the Paraná Flooded Savanna formed a second group (hereafter, “Delta” group). Insects collected from the Middle Paraná, situated between these two regions, were positioned between these two clusters in the PCA. Individuals from the Alto Paraná Atlantic Forests ecoregion, associated with the High Paraná River (hereafter, “Atlantic forest” group), comprised an isolated cluster. Finally, individuals from the potentially introduced population at Santa Teresita made up a separate fourth cluster.

Admixture analyzes using sNMF also indicated signals of spatial genetic structure (Figure 2B,C). When the number of ancestral populations was set to *K* = 3, the optimal *K* value according to the cross‐entropy criterion, sNMF recovered the three first groups from the PCA: Delta, Chaco and Atlantic Forest. Insects collected from the Middle Paraná showed mixed ancestry between the Delta and Chaco groups, while individuals from the Atlantic Forest group showed a high degree of admixture with the Chaco group. When K was set to 4, considering the results from the PCA, the same three groups remained consistent, while the Santa Teresita population emerged as a distinct fourth group. Genetic diversity was characterized for the 30 genotyped populations based on genome‐wide SNP data (Table S2). Overall, all indexes were homogeneous for all populations, indicating similar levels of genetic diversity. Most populations had no P_A_, except for Santa Teresita (Table S2).

### 3.3 Species Tree and Phylogenetic Relationships Among Populations

Phylogenetic relationships inferred with SNAPP showed that the Atlantic Forest and Chaco groups formed a sister clade, while the Delta group appeared as the most divergent lineage (Figure 2D). This topology supports a scenario where the Delta population diverged earlier, followed by a more recent split between the Atlantic Forest and Chaco populations. Pairwise F_ST_ values among the three main genetic groups were 0.038 between Delta and Chaco, 0.048 between Chaco and Atlantic Forest, and 0.062 between Delta and Atlantic Forest.

**FIGURE 3:**
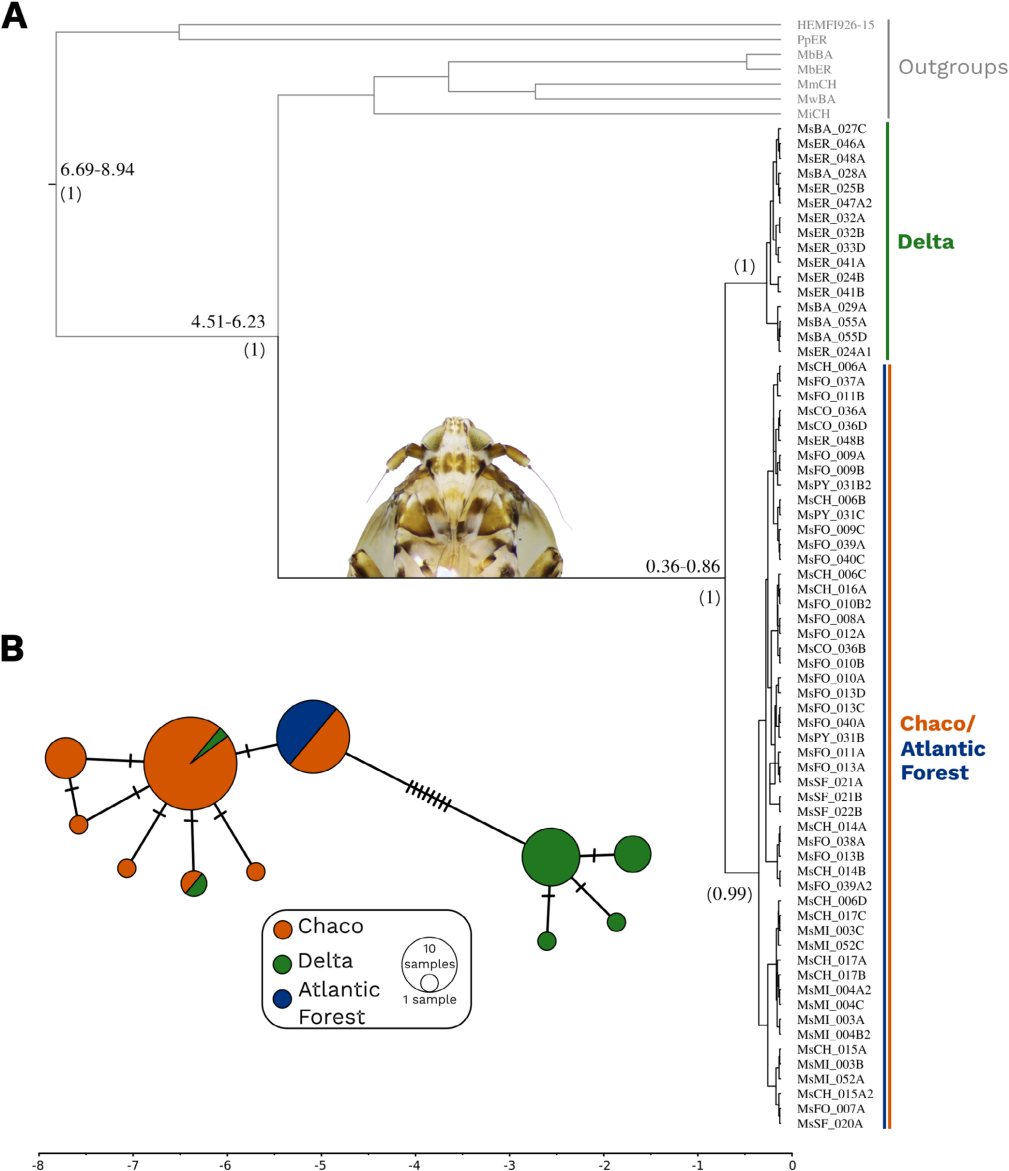
Mitochondrial genetic structure and phylogeny of *Megamelus scutellaris* . (A) Bayesian phylogeny inferred in BEAST2 using mtDNA. Node labels indicate estimated divergence times (in Mya) and posterior probabilities (in brackets); branch lengths are proportional to genetic divergence. Divergence time intervals are shown as 95% highest posterior density (HPD) intervals. Time scale is in millions of years ago (Mya). (B) Haplotype network based on mitochondrial data, showing two distinct haplogroups. Circle sizes represent haplotype frequencies, and colors correspond to nuclear genetic groups.

For mitochondrial data, a total of 68 individuals were sequenced for 658 bp of the COI gene, yielding 11 haplotypes distributed in two distinct haplogroups (Figure 3B). One was associated with northern populations of *M. scutellaris* , aligning with the Chaco and Atlantic Forest groups from nDNA data, while the other clustered haplotypes from southern populations, corresponding to the Delta and Santa Teresita groups. The Bayesian phylogeny inferred in BEAST2 based on mtDNA recovered the same two clades (Figure 3A). Furthermore, divergence time estimates suggested that *M. scutellaris* diverged from its closest relatives approximately 5.39 million years ago (Mya), while the split between the Delta and Chaco/Atlantic Forest clades occurred around 0.64 Mya (Figure 3A).

### 3.4 Demographic Inference

Alternative demographic scenarios were tested using coalescent‐based simulations in *fsc2*, based on a SFS constructed from 4302 biallelic and unlinked SNPs. The best‐supported model included a full post‐divergence migration matrix with asymmetric migration rates among the three genetic groups (Table S6). The second‐best model featured symmetric migration rates, while the least supported scenario assumed no migration between demes.

Under the best‐supported demographic model, the divergence between the Delta group and the most recent common ancestor of the Chaco and Atlantic Forest groups was estimated to have occurred approximately 5.9 million generations ago, while the split between the latter two lineages took place 97,000 generations ago (Table 1). Assuming a generational time of ~36 days (10 generations per year) at 25°C (May and Coetzee 2013), these events would date to approximately 0.59 and 0.01 Mya, respectively. Gene flow patterns among these groups suggest asymmetric migration dynamics. Migration rates between Chaco and Delta were relatively balanced, with similar values in both directions (Table 1). In contrast, gene flow between the Atlantic Forest and the other groups was markedly asymmetrical. Migration from the Atlantic Forest into the Delta was stronger than in the opposite direction, indicating predominantly north‐to‐south movement along the Paraná River. Similarly, gene flow from the Atlantic Forest into the Chaco was higher than vice versa, consistent with east‐to‐west dispersal patterns following the river's course (Table 1).

**TABLE 1:** Parameters inferred from coalescent simulations with *fsc2* under the best‐supported model (illustrated in Figure 1B) of divergence and gene flow among the three main genetic groups (POP0: Delta; POP1: Chaco; POP2: Atlantic Forest).

Parameter	Point estimate	95% CI
N_ANC1_	724,531	124,969–724,531
N_ANC2_	2,695,083	1,531,543–2,695,083
NPOP0	202,723	169,152–256,130
NPOP2	46,280	37,777–58,642
TDIV1	5,937,382	3,458,346–5,937,382
TDIV2	97,835	81,333–131,814
MIG01	3.33E‐05	2.61 × 10^−05^–3.5 × 10^−05^
MIG10	3.57E‐06	3.05 × 10^−06^–5.73 × 10^−06^
MIG02	4.01E‐10	3.84 × 10^−11^–2.40 × 10^−06^
MIG20	2.86E‐05	1.53 × 10^−05^–4.20 × 10^−05^
MIG12	6.42E‐10	1.43 × 10^−10^–4.60 × 10^−06^
MIG21	7.95E‐05	6.14 × 10^−04^–1.02 × 10^−04^

*Note:* The table presents point estimates along with the 95% confidence intervals (lower and upper bounds) for each parameter. These parameters include mutation‐scaled ancestral (N_ANC1_ and N_ANC2_) and contemporary (NPOP0 and NPOP2) effective population sizes, migration rates per generation (MIG01, MIG10, MIG02, MIG20, MIG12, and MIG21), and the timing of population divergence (TDIV1 and TDIV2). Time estimates are provided in generations. The effective population size of POP1 (NPOP1) was derived from nucleotide diversity and fixed in *fsc2* analyzes to facilitate the estimation of other parameters (see Methods for details).

### 3.5 Landscape Genomics

Genetic differentiation (F_ST_) was positively associated with geographic distance (IBD, resistance matrix based on a “flat” landscape), environmental distances between localities (IBE_CLIM_), host plant distribution (IBR_HOST_), and habitat resistance distances based on an ENM built from climatic variables (IBR_ENM_) (Figure 1D) when these variables were included alone in univariate models (Table S7). Models including IBE_CLIM_, IBR_ENM_, and IBD had the highest coefficients of determination (r^2^). The only variable that did not significantly explain genetic differentiation was surface freshwater occurrence (IBR_WATER_). When all significant variables were included in a single model and they were selected through a backward procedure, IBE_CLIM_ and IBR_ENM1_ retained significance and were selected for the final model (*r* ^2^ = 0.70) (Table 2).

**TABLE 2:** Results of the Multiple Matrix Regression with Randomization (MMRR) analysis assessing the relationship between genetic differentiation (*F*_ST_) and explanatory variables.

Model	*r* ^2^	Variable	β	*t*	*p*
Full model	0.70	IBD	0.07	0.54	0.812
		IBE_CLIM_	0.53	7.16	0.001
		IBR_HOST_	0.002	0.03	0.993
		IBR_ENM_	0.38	10.70	0.001
Backward variable selection					
Final model	0.70	IBE_CLIM_	0.59	17.10	0.001
		IBR_ENM1_	0.38	11.10	0.001

*Note:* The analysis began with a full model including four predictors: Geographic distance (IBD), environmental distance (IBE_CLIM_), host plant distribution (IBR_HOST_), and habitat resistance inferred from a climate‐based ecological niche model (IBR_ENM_). A backward selection procedure was applied, sequentially removing the least significant variable until only significant predictors remained. The final model retained IBE_CLIM_ and IBR_ENM_ (*r* ^2^ = 0.70). The table reports the coefficient of determination (*r* ^2^), regression coefficients (β), *t*‐values (*t*), and *p*‐values (*p*) for each model.

Consistent with the relevance of environmental distances (IBE_CLIM_), the PCA of the 15 CHELSA bioclimatic variables revealed clear clustering of sampling localities in environmental space (Figure S3). The three main genetic groups (Delta, Chaco, and Atlantic Forest) occupied distinct regions along the first two PCA axes, indicating environmental separation that mirrors the observed genetic structure. The environmental variables contributing most strongly to PC1 (49.2% of the variation) included temperature seasonality, annual temperature range, mean daily minimum temperature of the coldest month, and annual precipitation. PC2 (25.4% of the variation) was mainly driven by maximum air temperature of the warmest month and mean temperature of the warmest quarter. Variables were considered as major contributors when they showed both significant correlation with a given axis and a correlation coefficient above 0.8 (Table S8).

## 4 Discussion

In the present work, we combined nuclear genome‐wide SNP data and mitochondrial sequence data to investigate the genetic structure and evolutionary history of *Megamelus scutellaris* across its native range in Argentina and southern Paraguay. Both datasets support a deep divergence, with nuclear data further resolving a more recent split, ultimately revealing three main genetic groups (Chaco, Atlantic Forest, and Delta) broadly corresponding with major ecoregions and river basins. Landscape genomic analyzes show that genetic variation is significantly associated with climatic differences and landscape resistance, supporting models of isolation by environment and isolation by resistance. These findings not only shed light on the evolutionary history of *M. scutellaris* but also provide a valuable baseline for future assessments of its suitability as a biological control agent across environmentally diverse regions.

Analyzes of population structure based on nDNA consistently identified three main genetic lineages: Chaco, Atlantic Forest, and Delta. The most ancient divergence, between the Delta lineage and the most recent common ancestor of the Chaco and Atlantic Forest lineages, was consistent across markers, occurring approximately 0.59 Mya based on nDNA and ~0.64 Mya based on mtDNA. Such divergence likely reflects climatic, geomorphological, and ecological changes that took place during the Pleistocene, a period characterized by intensified glacial–interglacial cycles which dramatically reshaped South American landscapes, altering vegetation, river dynamics, freshwater connectivity, and sea levels (Panario and Gutiérrez 1999; Moraes et al. 2009). The estimated divergence between the Delta lineage and the ancestral Chaco–Atlantic Forest group may reflect the impact of major glacial and arid events during the Middle Pleistocene. In particular, the Coldest Pleistocene Glaciation (CPG, ~0.78 Mya) was associated with colder and drier conditions in the Pampean region (Soibelzon and Tonni 2009). These conditions likely disrupted hydrological connectivity and reduced the distribution of aquatic vegetation such as *P. crassipes* , which may have led to the isolation of *M. scutellaris* populations in the Lower Paraná floodplains, where the Delta lineage persisted and diverged, while northern populations remained in more stable refugia within the Chaco and Atlantic Forest regions. Moreover, sedimentological and paleomagnetic data from the Mesopotamian region indicate the development of swampy and lacustrine environments under cold and alternating humid and arid conditions during the early to middle Pleistocene (Formación Hernandarias, 0.8–1.3 Mya) (Aceñolaza 2004). These settings suggest that the Lower Paraná region may have retained suitable microhabitats for aquatic vegetation and associated fauna during periods of regional aridity, supporting the hypothesis that the Delta lineage of *M. scutellaris* persisted in southern refugia during glacial episodes.

Evidence of Pleistocene glacial cycles shaping genetic structure has also been found in other taxa in the region. For example, Brusquetti et al. (2019) found signals of population expansion of the frog *Leptodactylus bufonius* in the Chaco which correspond to the warmer and more humid interglacial period between the Greatest Patagonian Glaciation (GPG) and the CPG. Similarly, Langone et al. (2016) showed that major colonization events in the Pampean frog *Pseudopaludicola falcipes* also occurred during interglacial periods, suggesting a role for these phases in promoting lineage differentiation and long‐distance expansion, although no strong demographic shifts were detected. Divergence events during this period have also been recorded for species distributed in other seasonally deciduous forests and open vegetation formations in South América, including the cactophilic fly *Drosophila gouveai* (Moraes et al. 2009), the spider *Sicarius cariri* (Magalhaes et al. 2014), and the narrow‐billed woodcreeper *Lepidocolaptes angustirostris* (Rocha et al. 2020), highlighting the widespread impact of these climatic shifts across diverse environments and organisms.

On the other hand, the divergence between the Chaco and Atlantic Forest lineages of *M. scutellaris* was estimated to have occurred approximately 10 kya. This timing corresponds to the early Holocene, a transitional period following the Last Glacial Maximum, marked by increasing temperatures and the onset of more humid conditions in the Argentine Mesopotamia, which favored the development of fluvial networks and aquatic habitats (Iriondo 2010; Santa Cruz et al. 2020). Although this divergence likely reflects habitat fragmentation and range shifts associated with climatic dynamics, the precise correlation between these environmental transitions and the demographic patterns observed in *M. scutellaris* remains largely conjectural. Interestingly, a recent genomic study on *P. crassipes* , the host plant of *M. scutellaris* , reported mild genetic structuring between populations from the Pantanal and the upper Paraná basin in Brazil (da Cunha et al. 2022), suggesting that hydrological separation among these basins may contribute to genetic differentiation in aquatic taxa. Given that the Pantanal is associated with the Paraguay river (and thus the Chaco lineage), and the Upper Paraná basin with the Atlantic Forest lineage, these results parallel the genetic break observed in *M. scutellaris* . Notably, the downstream connection between the Upper Paraná and the Paraguay–Middle Paraná system was only established in the early Holocene (Arzamendia and Giraudo 2009). However, even after this connection, several studies have shown that species restricted to the Upper Paraná do not always extend into the Middle Paraná, indicating that biological connectivity may remain limited despite hydrological continuity (Arzamendia and Giraudo 2009). In this context, the divergence between Chaco and Atlantic Forest lineages may reflect a period of earlier isolation, possibly during the Last Glacial Maximum, followed by recent secondary contact, as evidenced by contemporary gene flow and admixture, particularly from the Atlantic Forest into Chaco.

In addition to the three main native lineages described above, the Santa Teresita population stands out as a noteworthy case. This population is located in the Humid Pampas ecoregion, restricted to an artificial water body, and does not occur within the river network that connects all native populations of neither *M. scutellaris* nor its host plant. Its geographic context, together with its genetic distinctiveness, suggests that it represents an unintentional human‐mediated introduction rather than a lineage that is part of the natural evolutionary history of the species in the Plata basin. For this reason, we excluded Santa Teresita from demographic analyzes aimed at reconstructing divergence among native lineages, as well as from landscape genomic analyzes since its geographic isolation and distinct environmental context would introduce noise and alter the set of retained predictor variables. Despite this, Santa Teresita remains of considerable applied and evolutionary interest. It represents the southernmost known occurrence of *M. scutellaris* , persisting in colder conditions than its native range, and its long‐term establishment outside the river system may reflect mechanisms such as phenotypic plasticity or local adaptation. Understanding the factors that enable its persistence could provide valuable insights for both biological control and evolutionary ecology, and warrants targeted investigation in future studies.

Our demographic analyzes supported a model of contemporary asymmetric gene flow between lineages with higher migration rates from the Atlantic Forest into the Chaco region. This directionality aligns with current hydrological connectivity, as the High Paraná River flows westward from the Atlantic Forest into the Chaco region, potentially facilitating post‐divergence dispersal on plant mats and maintaining genetic connectivity. However, the Atlantic Forest group remains clearly isolated in PCA and sNMF analyzes, despite the identified gene flow. This may be because the High Paraná River and its basin harbor relatively sparse populations of water hyacinth (Bonetto et al. 1986). In line with this, *M. scutellaris* was not detected along most of its course, despite extensive sampling efforts conducted for this study. In contrast, gene flow between Chaco and Delta was more balanced, but slightly higher from Delta into Chaco. Although there is a general north‐to‐south directionality in the flow of major rivers, with Chaco associated with the Paraguay River and Delta with the Middle and Lower Paraná, the presence of extensive floodplains and interconnected water bodies where water hyacinth thrives (Carignan and Neiff 1992) likely facilitates gene flow in both directions. These patterns of gene flow are consistent with the levels of genetic differentiation observed: pairwise F_ST_ values were lowest between Delta and Chaco (0.038), intermediate between Chaco and Atlantic Forest (0.048), and highest between Delta and Atlantic Forest (0.062). Interestingly, although the divergence between Chaco and Atlantic Forest occurred more recently, genetic differentiation between them is greater than between Delta and Chaco, likely reflecting the combined effects of ecological separation and limited effective gene flow despite hydrological connectivity.

In agreement with the inferred gene flow, *M. scutellaris* samples from the Middle Paraná River exhibited mixed nuclear ancestry between Chaco and Delta, alongside mitochondrial haplotypes from both major clades. These findings support the role of this region as a contact or secondary mixing zone. This area overlaps with the Espinal ecoregion, a transitional zone between the Chaco and Pampa, which has been identified as a biogeographic boundary in several taxa. In insects such as the cactus moth *Cactoblastis cactorum* (Berg), evidence of nuclear admixture and shared mitochondrial haplotypes was detected across the Chaco–Pampa contact zone (Poveda‐Martínez et al. 2023; Andraca‐Gómez et al. 2024). These parallels suggest that the Espinal may have contributed to the genetic landscape observed in *M. scutellaris* by modulating dispersal and connectivity.

Landscape genomic analyzes revealed a strong association between genomic variation and climatic heterogeneity across the range of *M. scutellaris* . Genetic differentiation was most strongly explained by climatic dissimilarity among localities and by climate‐based landscape resistance, while resistance surfaces based on host plant distribution or geographic distance contributed less once evaluated in combination. A principal component analysis of the bioclimatic variables revealed clear environmental separation among sampling localities, with each of the three main genetic lineages (Delta, Chaco, and Atlantic Forest) occupying distinct climatic spaces that broadly correspond to distinct ecoregions. Together, these findings suggest that climatic suitability and climate‐driven connectivity may have contributed to the observed patterns of genomic differentiation in *M. scutellaris* , although it cannot be ruled out that part of this signal reflects historical isolation rather than local adaptation.

In this study, we established that the two populations of *M. scutellaris* exported for the biological control of *P. crassipes* belong to distinct genetic lineages: while the insects first introduced to the U.S. and subsequently to South Africa originated from the Delta lineage, the second introduction to the U.S. corresponds to the Chaco lineage. Genetic approaches have also been applied in other biological control systems to characterize population structure and genetic variation in control agents. For example, species delimitation analyzes in South American phorid flies identified multiple lineages with potentially different effectiveness against fire ants (Sánchez‐Restrepo et al. 2020). In *Hypogeococcus* Rau mealybugs, combined genetic and morphological analyzes clarified the identity of the population introduced to Australia, revealing it belongs to the *Hypogeococcus pungens* Granara de Willink species complex rather than *Hypogeococcus festerianus* (Lizer & Trelles), and confirming its compatibility with the host plant despite prior taxonomic confusion (Ezeh et al. 2023). Likewise, recent work on *Lysathia* Bechyné beetles revealed that the population successfully used in South Africa against *Myriophyllum aquaticum* (Vell.) Verdc represents a distinct, previously undescribed species native to Argentina (Faltlhauser et al. 2025). Together, these examples underscore the value of characterizing genetic structure in the native range of biological control agents. Such analyzes can reveal cryptic diversity, help avoid the inadvertent introduction of undesired taxa, and provide a foundation for future work aimed at identifying lineage‐specific traits that may influence establishment, performance, or environmental tolerance. In the case of *M. scutellaris* , recognizing that different introduction events involved distinct lineages provides a framework for reinterpreting past biocontrol outcomes and for guiding future efforts, including the evaluation of lineage‐specific responses to environmental conditions and the monitoring of evolutionary trajectories in introduced populations.

Additionally, our results reveal an association between genetic and climatic distances, which may reflect population‐specific responses to environmental variation. This pattern is consistent with previous studies reporting differences in performance between individuals from the Delta and Chaco lineages under different temperature regimes in laboratory experiments (Foley et al. 2016). Although several studies in biological control have incorporated biogeographic approaches and ecological niche modeling to inform decision‐making (Kriticos et al. 2021; Zhao et al. 2023), few have integrated landscape genomic analyzes to evaluate how genetic structure and environmental heterogeneity may jointly shape the adaptive potential of biological control agents across their native ranges. Rather than detecting adaptation directly, our framework sets the stage for future work explicitly testing local adaptation and evaluating how different lineages respond to contrasting climatic conditions. Such data can inform climate‐matching approaches when selecting or re‐evaluating candidate populations for introduction, ultimately supporting the long‐term success of water hyacinth biocontrol programs across diverse regions.

Future research should explicitly address the genetic and ecological trajectories of introduced populations, particularly those established in South Africa and the United States. Comparing their responses to local environmental conditions over several generations could provide critical insights into the predictability of establishment, spread, and impact. Such studies will also contribute to broader invasion biology frameworks by contrasting native and introduced contexts, while informing the refinement of classical biocontrol strategies based on genetic and ecological compatibility.

## Funding

This work was supported by Agricultural Research Service, Consejo Nacional de Investigaciones Científicas y Técnicas, and Fundación para el Estudio de Especies Invasivas (FuEDEI).

## Conflicts of Interest

The authors declare no conflicts of interest.

## Supporting information


**Table S1:** Population sampling of *Megamelus scutellaris* .
**Table S2:** Summary statistics of genetic diversity from sampling localities.
**Table S3:** CHELSA variables used for landscape genomics analyzes.
**Figure S1:** PCA of environmental variables for IBE analysis.
**Figure S2:** Ecological niche model (ENM) for *Pontederia crassipes*.
**Table S4:** Sequencing and assembly summary metrics.
**Table S5:** Number of SNPs retained after each quality filtering step.
**Table S6:** Comparison between alternative demographic models.
**Table S7:** Results of univariate matrix regressions with randomization.
**Figure S3:** Climatic variation across sampling sites.
**Table S8:** Pearson correlation coefficients and associated *p*‐values for each CHELSA bioclimatic variable.

## Data Availability

The data that support the findings of this study are openly available in Figshare at https://doi.org/10.6084/m9.figshare.28436882. The mtDNA haplotypes are available in Genbank under accession numbers PV190212‐PV190271. Raw sequencing reads have been deposited in NCBI under BioProject PRJNA1222173.
