# Supplementary material for: Landscape Genomics and Evolutionary History of Megamelus scutellaris, a Biocontrol Agent of the Invasive Water Hyacinth (Pontederia crassipes)
Source: Evol Appl. 2026 Feb 18;19(2):e70208. doi: 10.1111/eva.70208 (PMC12916152; doi:10.1111/eva.70208)
Supplement: Supplementary file 1 — Table S1: Population sampling of Megamelus scutellaris . Table S2: Summary statistics of genetic diversity from sampling localities. Table S3: CHELSA variables used for landscape genomics analyzes. Figure S1: PCA of environmental variables for IBE analysis. Figure S2: Ecological niche model (ENM) for Pontederia crassipes. Table S4: Sequencing and assembly summary metrics. Table S5: Number of SNPs retained after each quality filtering step. Table S6: Comparison between alternative demographic models. Table S7: Results of univariate matrix regressions with randomization. Figure S3: Climatic variation across sampling sites. Table S8: Pearson correlation coefficients and associated p‐values for each CHELSA bioclimatic variable. [file EVA-19-e70208-s001.docx]

Supplementary Information for:

Landscape genomics and evolutionary history of *Megamelus scutellaris*, biocontrol agent of the invasive water hyacinth (*Pontederia crassipes*)

Nicolas A. Salinas, Daniel Poveda-Martínez, Marcela S. Rodriguero, Melissa Smith, María E. Brentassi & Alejandro J. Sosa

**Table of contents**

**Table S1.** Population sampling of *Megamelus scutellaris*.

**Table S2.** Summary statistics of genetic diversity from sampling localities.

**Table S3.** CHELSA variables used for landscape genomics analyses

**Figure S1.** PCA of environmental variables for IBE analysis.

**Figure S2.** Ecological niche model (ENM) for *Pontederia crassipes.*

**Table S4.** Sequencing and assembly summary metrics

**Table S5.** Number of SNPs retained after each quality filtering step.

**Table S6.** Comparison between alternative demographic models.

**Table S7.** Results of univariate matrix regressions with randomization.

**Figure S3**. Climatic variation across sampling sites.

**Table S8.** Pearson correlation coefficients and associated p-values for each CHELSA bioclimatic variable

**Table S1.** Population sampling of *Megamelus scutellaris*. Information on sampling sites used in genomic analyses, including sampling code, country, province, site name, latitude and longitude, and collection date.

| **Site code** | **Country** | **Province** | **Site name** | **Latitude** | **Longitude** | **Date** |
| --- | --- | --- | --- | --- | --- | --- |
| MsMI_003 | Argentina | Misiones | Garupá | -27.4729 | -55.8048 | 1 Dec 2021 |
| MsMI_004 | Argentina | Misiones | Garupá | -27.4792 | -55.7933 | 1 Dec 2021 |
| MsMI_052 | Argentina | Misiones | San Ignacio - Río Yaberiby | -27.2841 | -55.5347 | 27 Nov 2022 |
| MsCO_036 | Argentina | Corrientes | Ramada Paso - Camping Curuzú Jaime | -27.3633 | -58.2969 | 15 May 2022 |
| MsCH_006 | Argentina | Chaco | San Martin - Laguna El Tigre | -26.5591 | -59.3158 | 3 Dec 2021 |
| MsCH_017 | Argentina | Chaco | Fortín Chajá | -27.0467 | -59.7368 | 6 Dec 2021 |
| MsFO_007 | Argentina | Formosa | Colonia Pres. Yrigoyen | -26.1880 | -58.7726 | 3 Dec 2021 |
| MsFO_008 | Argentina | Formosa | Formosa - Regimiento Infanteria | -26.1590 | -58.1524 | 4 Dec 2021 |
| MsFO_009 | Argentina | Formosa | Ruta 2 - Riacho He He | -25.4379 | -58.2545 | 4 Dec 2021 |
| MsFO_010 | Argentina | Formosa | Ruta 2 - Laguna Blanca | -25.1624 | -58.2346 | 4 Dec 2021 |
| MsFO_011 | Argentina | Formosa | Ruta 3 - Camino a Pirané | -25.1894 | -58.5943 | 4 Dec 2021 |
| MsFO_012 | Argentina | Formosa | Pirané | -25.7334 | -59.0945 | 4 Dec 2021 |
| MsCH_014 | Argentina | Chaco | Fortín Lavalle | -25.6906 | -60.1505 | 5 Dec 2021 |
| MsCH_015 | Argentina | Chaco | Río Bermejito | -25.6302 | -60.2677 | 5 Dec 2021 |
| MsCH_016 | Argentina | Chaco | La Pelole - Rio Bermejito | -25.6021 | -60.2833 | 5 Dec 2021 |
| MsSF_020 | Argentina | Santa Fe | Villa Ocampo | -28.5056 | -59.2610 | 6 Dec 2021 |
| MsSF_021 | Argentina | Santa Fe | Arroyo Curupí - Puerto Reconquista | -29.2302 | -59.5870 | 7 Dec 2021 |
| MsSF_022 | Argentina | Santa Fe | Bajada lanchas - Cerca San Javier | -30.4277 | -59.9436 | 7 Dec 2021 |
| MsSF_023 | Argentina | Santa Fe | Saladero Mariano Cabal | -30.8854 | -60.0346 | 7 Dec 2021 |
| MsER_033 | Argentina | Entre Ríos | Piedras Blancas - Camping El Saucedal | -31.1931 | -59.9650 | 12 May 2022 |
| MsER_024 | Argentina | Entre Ríos | Ibicuy - Camping El Paraiso | -33.8650 | -58.8831 | 5 Feb 2022 |
| MsER_025 | Argentina | Entre Ríos | Ibicuy - Camping El Paraiso 2 | -33.8735 | -58.8969 | 5 Feb 2022 |
| MsBA_026 | Argentina | Buenos Aires | San Pedro - Luz y Fuerza | -33.6811 | -59.6477 | 6 Feb 2022 |
| MsBA_027 | Argentina | Buenos Aires | San Pedro - Camping Municipal | -33.6706 | -59.6594 | 6 Feb 2022 |
| MsBA_028 | Argentina | Buenos Aires | Otamendi | -34.0808 | -58.8048 | 25 Feb 2022 |
| MsBA_029 | Argentina | Buenos Aires | Arroyo Pescado | -34.2655 | -58.9011 | 25 Mar 2022 |
| MsBA_054 | Argentina | Buenos Aires | Tigre - La Aguada | -34.3090 | -58.5996 | 8 Dec 2022 |
| MsBA_057 | Argentina | Buenos Aires | Arroyo El Diablo - Delta | -34.2627 | -58.4355 | 10 Feb 2023 |
| MsBA_056 | Argentina | Buenos Aires | Río Molina - Delta | -33.9942 | -58.8733 | 10 Feb 2023 |
| MsPY_031 | Paraguay | Pte. Hayes | Villa Hayes | -25.1446 | -57.5558 | 7 Apr 2022 |
| MsFO_037 | Argentina | Formosa | Las Lomitas - Vertedero I | -24.3811 | -60.3246 | 26 May 2022 |
| MsFO_038 | Argentina | Formosa | Las Lomitas - Vertedero II | -24.3614 | -60.3081 | 26 May 2022 |
| MsFO_039 | Argentina | Formosa | Las Lomitas - Vertedero III | -24.3390 | -60.2902 | 26 May 2022 |
| MsFO_040 | Argentina | Formosa | Pozo del Tigre - Río Monte Lindo | -24.8624 | -60.2897 | 28 May 2022 |
| MsFO_013 | Argentina | Formosa | Ibarreta | -25.2352 | -59.8051 | 5 Dec 2021 |
| MsER_041 | Argentina | Entre Ríos | Concordia - Arroyo Manzores | -31.3996 | -58.0027 | 30 May 2022 |
| MsER_048 | Argentina | Entre Ríos | Colonia Ayuí | -31.1891 | -58.0243 | 26 Nov 2022 |
| MsER_046 | Argentina | Entre Ríos | Villa Paranacito - Río Paranacito | -33.7165 | -58.6609 | 25 Nov 2022 |
| MsER_047 | Argentina | Entre Ríos | Villa Paranacito - Arroyo | -33.6622 | -58.7232 | 25 Nov 2022 |
| MsER_032 | Argentina | Entre Ríos | Ruta 14 - km 4-5 | -33.4624 | -58.8024 | 11 May 2022 |
| MsBA_055 | Argentina | Buenos Aires | Santa Teresita | -36.5490 | -56.7170 | 7 Jan 2023 |

**Table S2.** Summary statistics of genetic diversity of *M. scutellaris* sampling localities, including samples per locality (N), observed and expected heterozygosity (H_O_ and H_E_, respectively), inbreeding coefficient (F_IS_) and private alleles (P_A_).

| **Population** | **Sites codes** | **N** | **H_O_** | **H_E_** | **F_IS_** | **F_IS_ SE** | **P_A_** |
| --- | --- | --- | --- | --- | --- | --- | --- |
| Garupa | MsMI_003 - MsMI_004 | 9 | 0.162 | 0.192 | 0.111 | 0.01145 | 0 |
| San Ignacio | MsMI_052 | 3 | 0.182 | 0.169 | 0.043 | 0.00449 | 0 |
| Ramada Paso | MsCO_036 | 4 | 0.184 | 0.184 | 0.060 | 0.00541 | 0 |
| San Martín | MsCH_006 - MsCH_017 | 5 | 0.180 | 0.189 | 0.070 | 0.00668 | 0 |
| Yrigoyen | MsFO_007 | 3 | 0.185 | 0.174 | 0.051 | 0.00443 | 0 |
| Regimiento Infantería | MsFO_008 | 3 | 0.181 | 0.172 | 0.050 | 0.00412 | 0 |
| Riacho He He | MsFO_009 | 3 | 0.180 | 0.172 | 0.054 | 0.00443 | 0 |
| Laguna Blanca | MsFO_010 | 3 | 0.184 | 0.174 | 0.052 | 0.00446 | 0 |
| Ruta 3 | MsFO_011 | 3 | 0.180 | 0.169 | 0.050 | 0.00504 | 0 |
| Fortín Lavalle | MsCH_014 | 4 | 0.187 | 0.186 | 0.056 | 0.00538 | 0 |
| Río Bermejito | MsCH_015 - MsCH_016 | 4 | 0.184 | 0.187 | 0.066 | 0.00535 | 0 |
| Villa Ocampo | MsSF_020 | 3 | 0.182 | 0.174 | 0.056 | 0.00488 | 0 |
| Puerto Reconquista | MsSF_021 | 3 | 0.166 | 0.163 | 0.064 | 0.00572 | 0 |
| Mariano Cabal | MsSF_023 | 3 | 0.167 | 0.165 | 0.067 | 0.0055 | 0 |
| Piedras Blancas | MsER_033 | 4 | 0.168 | 0.180 | 0.081 | 0.00645 | 0 |
| Ibicuy | MsER_024 - MsER_025 | 6 | 0.172 | 0.192 | 0.093 | 0.0092 | 0 |
| San Pedro | MsBA_026 - MsBA_027 | 6 | 0.187 | 0.198 | 0.071 | 0.00641 | 0 |
| Otamendi | MsBA_028 | 3 | 0.179 | 0.169 | 0.051 | 0.00486 | 0 |
| Arroyo Pescado | MsBA_029 | 4 | 0.179 | 0.183 | 0.066 | 0.00504 | 0 |
| El Diablo | MsBA_057 | 3 | 0.159 | 0.158 | 0.068 | 0.00648 | 0 |
| Río Molina | MsBA_056 | 3 | 0.178 | 0.173 | 0.060 | 0.0045 | 0 |
| Paraguay | MsPY_031 | 3 | 0.185 | 0.170 | 0.042 | 0.00478 | 0 |
| La Estrella | MsFO_037 - MsFO_038 - MsFO_039 | 4 | 0.169 | 0.176 | 0.079 | 0.00825 | 0 |
| Pozo del Tigre | MsFO_040 | 3 | 0.177 | 0.168 | 0.054 | 0.00513 | 0 |
| Ibarreta | MsFO_013 | 4 | 0.179 | 0.183 | 0.066 | 0.00567 | 0 |
| Concordia | MsER_041 | 4 | 0.176 | 0.179 | 0.063 | 0.00518 | 0 |
| Colonia Ayuí | MsER_048 | 4 | 0.170 | 0.180 | 0.076 | 0.00535 | 0 |
| Paranacito | MsER_046 - MsER_047 | 5 | 0.175 | 0.186 | 0.076 | 0.00721 | 0 |
| Ceibas | MsER_032 | 3 | 0.173 | 0.169 | 0.060 | 0.00467 | 0 |
| Santa Teresita | MsBA_055 | 8 | 0.155 | 0.178 | 0.086 | 0.00907 | 3 |

**Table S3.** CHELSA variables used for landscape genomics analyses. Table shows variable code and its description.

| **CHELSA variable code** | **Variable description** |
| --- | --- |
| bio1 | mean annual air temperature |
| bio2 | mean diurnal air temperature range |
| bio3 | isothermality |
| bio4 | temperature seasonality |
| bio5 | mean daily maximum air temperature of the warmest month |
| bio6 | mean daily minimum temperature of the coldest month |
| bio7 | annual range of air temperature |
| bio10 | mean daily mean air temperatures of the warmest quarter |
| bio11 | mean daily mean air temperatures of the coldest quarter |
| bio12 | annual precipitation amount |
| bio13 | precipitation amount of the wettest month |
| bio14 | precipitation amount of the driest month |
| bio15 | precipitation seasonality |
| bio16 | mean monthly precipitation amount of the wettest quarter |
| bio17 | mean monthly precipitation amount of the driest quarter |


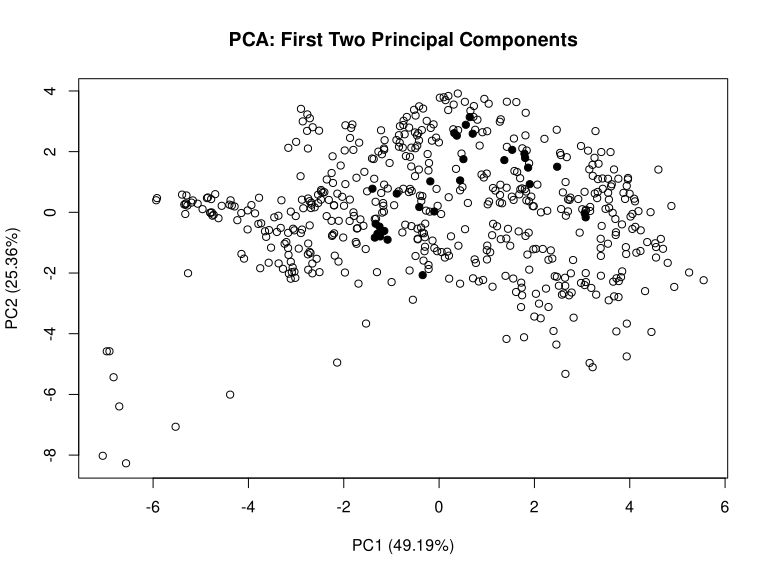


**Figure S1.** Principal Component Analysis (PCA) of environmental variables across the study area. The first two principal components (PC1 and PC2) are shown, summarizing the main axes of environmental variation. Black dots represent the 30 sampling sites, while open circles denote the 500 randomly sampled points used to account for broader environmental variability.


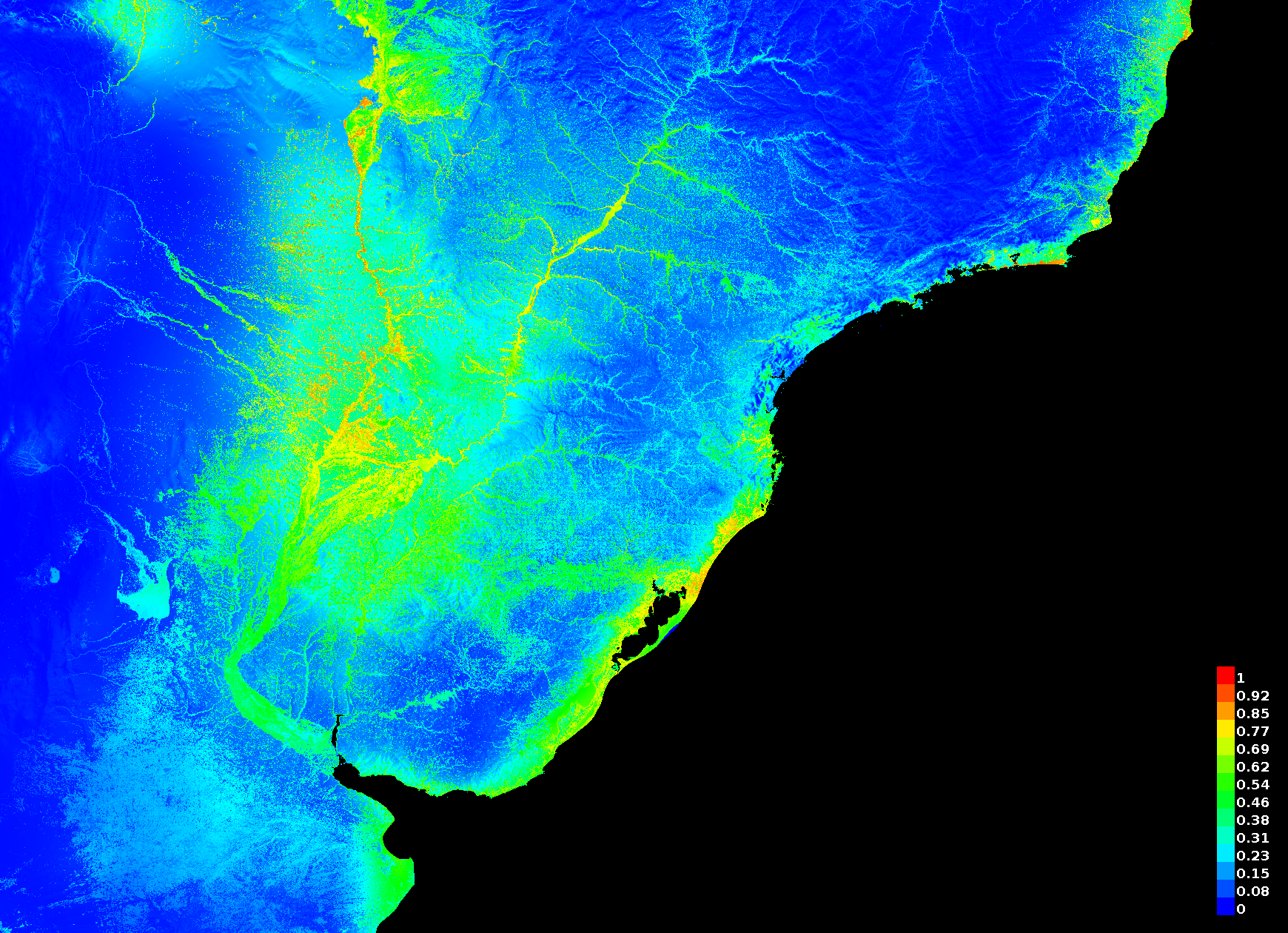


**Figure S2.** Ecological niche model (ENM) for *Pontederia crassipes*, based on CHELSA bioclimatic variables (Bio 2, 3, 5, 13, 15) and freshwater occurrence data. Warmer colors indicate higher habitat suitability for the species.

**Table S4.** Sequencing and assembly summary metrics for each of the 125 individuals included in this study. The table includes per-sample coverage, number of raw reads, reads retained after filtering, reference-mapped reads, and number of loci in the final assembly.

|  | **Coverage** | **Raw reads** | **Reads passed filter** | **Mapped reads** | **loci in assembly** |
| --- | --- | --- | --- | --- | --- |
| MsBA_026_Atrimmed | 10796 | 2108279 | 2052004 | 1156084 | 10796 |
| MsBA_026_Btrimmed | 10238 | 2428787 | 2379634 | 1245796 | 10238 |
| MsBA_026_Ctrimmed | 10601 | 1974537 | 1917085 | 1005131 | 10601 |
| MsBA_027_Atrimmed | 10780 | 2848116 | 2756862 | 1447928 | 10780 |
| MsBA_027_Btrimmed | 10517 | 2065690 | 2012831 | 1127977 | 10517 |
| MsBA_027_Ctrimmed | 11510 | 2350265 | 2302520 | 1372263 | 11510 |
| MsBA_028_Atrimmed | 10410 | 2593743 | 2561619 | 1525902 | 10410 |
| MsBA_028_Btrimmed | 9837 | 1939687 | 1887614 | 1005694 | 9837 |
| MsBA_028_Ctrimmed | 10807 | 2230139 | 2172283 | 1223820 | 10807 |
| MsBA_029_Atrimmed | 10872 | 2216469 | 2175234 | 1257573 | 10872 |
| MsBA_029_Btrimmed | 10643 | 1962940 | 1919872 | 1099559 | 10643 |
| MsBA_029_Ctrimmed | 10827 | 2832801 | 2776252 | 1520942 | 10827 |
| MsBA_029_Etrimmed | 10706 | 2363808 | 2310309 | 1322137 | 10706 |
| MsBA_054_A1trimmed | 8256 | 1455155 | 1453164 | 807416 | 8256 |
| MsBA_054_A2trimmed | 11001 | 2315977 | 2262969 | 1279281 | 11001 |
| MsBA_055_A2trimmed | 10619 | 3173316 | 3073624 | 1600429 | 10619 |
| MsBA_055_Atrimmed | 10684 | 2269627 | 2238651 | 1208722 | 10684 |
| MsBA_055_B2trimmed | 10948 | 2779488 | 2694415 | 1360599 | 10948 |
| MsBA_055_Btrimmed | 10394 | 2390429 | 2336134 | 1208698 | 10394 |
| MsBA_055_Ctrimmed | 11245 | 2784804 | 2716057 | 1585109 | 11245 |
| MsBA_055_D2trimmed | 10961 | 2524307 | 2486521 | 1488031 | 10961 |
| MsBA_055_Dtrimmed | 9376 | 2353825 | 2310964 | 1244127 | 9376 |
| MsBA_055_E2trimmed | 10516 | 3564518 | 3493634 | 1773139 | 10516 |
| MsBA_056_A1trimmed | 11234 | 2361980 | 2311776 | 1303901 | 11234 |
| MsBA_056_A2trimmed | 10133 | 2227302 | 2171165 | 1193242 | 10133 |
| MsBA_056_A3trimmed | 10212 | 2367022 | 2329801 | 1231237 | 10212 |
| MsBA_057_A1trimmed | 7271 | 1532009 | 1524285 | 820815 | 7271 |
| MsBA_057_A2trimmed | 10227 | 2274554 | 2231646 | 1216403 | 10227 |
| MsBA_057_A3trimmed | 9656 | 1888985 | 1851911 | 1026766 | 9656 |
| MsCH_006_Ctrimmed | 10399 | 2641148 | 2618723 | 1539935 | 10399 |
| MsCH_006_Dtrimmed | 10377 | 3055216 | 3007375 | 1780639 | 10377 |
| MsCH_014_Atrimmed | 10377 | 2848800 | 2775225 | 1592918 | 10377 |
| MsCH_014_Btrimmed | 11075 | 3429204 | 3315811 | 1843502 | 11075 |
| MsCH_014_Ctrimmed | 10548 | 2624675 | 2570327 | 1417610 | 10548 |
| MsCH_014_Dtrimmed | 10452 | 2697559 | 2629664 | 1481808 | 10452 |
| MsCH_015_A2trimmed | 10543 | 4169651 | 4037267 | 2010345 | 10543 |
| MsCH_015_A3trimmed | 11240 | 4091474 | 3977433 | 2110687 | 11240 |
| MsCH_015_Atrimmed | 10253 | 3033396 | 2966994 | 1466716 | 10253 |
| MsCH_016_A2trimmed | 10674 | 2904561 | 2817691 | 1630185 | 10674 |
| MsCH_017_Atrimmed | 9771 | 2672953 | 2650889 | 1535853 | 9771 |
| MsCH_017_Btrimmed | 10978 | 2787468 | 2715295 | 1531016 | 10978 |
| MsCH_017_Ctrimmed | 10171 | 2714409 | 2634434 | 1437624 | 10171 |
| MsCO_036_A2trimmed | 11080 | 3034331 | 2940189 | 1499762 | 11080 |
| MsCO_036_Atrimmed | 10978 | 2763015 | 2677194 | 1478784 | 10978 |
| MsCO_036_Btrimmed | 9458 | 2747179 | 2692232 | 1381729 | 9458 |
| MsCO_036_Dtrimmed | 11368 | 2462133 | 2405484 | 1417562 | 11368 |
| MsER_024_Atrimmed | 9911 | 2128334 | 2095084 | 1162254 | 9911 |
| MsER_024_Btrimmed | 8912 | 2475873 | 2431013 | 1079588 | 8912 |
| MsER_024_Ctrimmed | 10080 | 2448627 | 2412510 | 1312405 | 10080 |
| MsER_025_Atrimmed | 10465 | 2763606 | 2725359 | 1531960 | 10465 |
| MsER_025_B2trimmed | 9303 | 2272138 | 2246171 | 1056583 | 9303 |
| MsER_025_Btrimmed | 8836 | 2006815 | 1987177 | 843126 | 8836 |
| MsER_032_Atrimmed | 10768 | 2681908 | 2629748 | 1642001 | 10768 |
| MsER_032_B2trimmed | 10547 | 2217423 | 2174956 | 1198676 | 10547 |
| MsER_032_Btrimmed | 9905 | 2128803 | 2096053 | 1189016 | 9905 |
| MsER_033_B2trimmed | 9213 | 1747366 | 1706310 | 868377 | 9213 |
| MsER_033_Btrimmed | 10012 | 2230588 | 2180870 | 1275552 | 10012 |
| MsER_033_Ctrimmed | 10216 | 2482795 | 2416118 | 1258409 | 10216 |
| MsER_033_Dtrimmed | 10234 | 3012327 | 2934770 | 1681054 | 10234 |
| MsER_041_Atrimmed | 11133 | 3248531 | 3193271 | 1531877 | 11133 |
| MsER_041_B2trimmed | 10235 | 2492771 | 2444287 | 1291740 | 10235 |
| MsER_041_Btrimmed | 10490 | 2220433 | 2181519 | 1154682 | 10490 |
| MsER_041_Ctrimmed | 11060 | 2373827 | 2306188 | 1287438 | 11060 |
| MsER_046_Atrimmed | 10901 | 2463169 | 2414025 | 1314834 | 10901 |
| MsER_046_Btrimmed | 8157 | 1463064 | 1459688 | 781042 | 8157 |
| MsER_046_Ctrimmed | 10392 | 2518983 | 2453806 | 1264712 | 10392 |
| MsER_047_A1trimmed | 10285 | 2283229 | 2216631 | 1156554 | 10285 |
| MsER_047_A2trimmed | 10655 | 2132215 | 2082904 | 1096308 | 10655 |
| MsER_048_A2trimmed | 10975 | 3019920 | 2963020 | 1620410 | 10975 |
| MsER_048_Atrimmed | 10583 | 2321908 | 2273984 | 1206487 | 10583 |
| MsER_048_B2trimmed | 10075 | 2898039 | 2829711 | 1642514 | 10075 |
| MsER_048_Btrimmed | 10797 | 2808592 | 2715790 | 1729067 | 10797 |
| MsFO_007_Atrimmed | 10578 | 3325031 | 3246890 | 1740860 | 10578 |
| MsFO_007_B2trimmed | 10441 | 2815289 | 2784470 | 1637217 | 10441 |
| MsFO_007_Btrimmed | 10749 | 3291942 | 3203007 | 1578990 | 10749 |
| MsFO_008_A2trimmed | 10779 | 2888735 | 2839718 | 1503433 | 10779 |
| MsFO_008_Atrimmed | 11162 | 2406440 | 2342198 | 1320017 | 11162 |
| MsFO_008_Btrimmed | 10286 | 2204661 | 2125631 | 1029690 | 10286 |
| MsFO_009_Atrimmed | 11086 | 3592917 | 3508605 | 1825366 | 11086 |
| MsFO_009_Btrimmed | 10831 | 3411777 | 3351158 | 1989109 | 10831 |
| MsFO_009_Ctrimmed | 10146 | 2617355 | 2601760 | 1606865 | 10146 |
| MsFO_010_Atrimmed | 10158 | 3558403 | 3479910 | 1922849 | 10158 |
| MsFO_010_B2trimmed | 10925 | 3658723 | 3549816 | 2011272 | 10925 |
| MsFO_010_Btrimmed | 10772 | 3543154 | 3482161 | 1909010 | 10772 |
| MsFO_011_A2trimmed | 8782 | 2213128 | 2181811 | 1240018 | 8782 |
| MsFO_011_Atrimmed | 10994 | 2899910 | 2848136 | 1811023 | 10994 |
| MsFO_011_Btrimmed | 10743 | 3756162 | 3666058 | 1779365 | 10743 |
| MsFO_012_Atrimmed | 10251 | 3261712 | 3190152 | 1627998 | 10251 |
| MsFO_012_Ctrimmed | 11287 | 4532099 | 4417219 | 2528473 | 11287 |
| MsFO_013_Atrimmed | 10612 | 3854815 | 3779366 | 1678624 | 10612 |
| MsFO_013_Btrimmed | 9502 | 3627048 | 3576192 | 2018488 | 9502 |
| MsFO_013_Ctrimmed | 10489 | 3241855 | 3164190 | 1674899 | 10489 |
| MsFO_013_Dtrimmed | 10423 | 2918176 | 2873110 | 1506873 | 10423 |
| MsFO_037_Atrimmed | 10756 | 3163764 | 3078313 | 1574187 | 10756 |
| MsFO_038_Atrimmed | 8121 | 1274935 | 1271096 | 744235 | 8121 |
| MsFO_039_A2trimmed | 10670 | 2890785 | 2793303 | 1456612 | 10670 |
| MsFO_039_Atrimmed | 7194 | 1480079 | 1474356 | 830801 | 7194 |
| MsFO_040_Atrimmed | 10743 | 2662061 | 2617845 | 1461162 | 10743 |
| MsFO_040_Btrimmed | 10902 | 2805505 | 2739148 | 1429593 | 10902 |
| MsFO_040_Ctrimmed | 8806 | 1690405 | 1675253 | 874312 | 8806 |
| MsMI_003_A_reptrimmed | 9208 | 3341508 | 3303303 | 1620672 | 9208 |
| MsMI_003_Atrimmed | 8657 | 2262650 | 2252751 | 1133823 | 8657 |
| MsMI_003_Btrimmed | 10071 | 2826873 | 2771091 | 1430374 | 10071 |
| MsMI_003_Ctrimmed | 9670 | 2403149 | 2348900 | 1194883 | 9670 |
| MsMI_004_A2trimmed | 10438 | 1968790 | 1925747 | 1087018 | 10438 |
| MsMI_004_Atrimmed | 9624 | 2838987 | 2810751 | 1464219 | 9624 |
| MsMI_004_B2trimmed | 10320 | 2922665 | 2865066 | 1504517 | 10320 |
| MsMI_004_Btrimmed | 10262 | 2729652 | 2666443 | 1529923 | 10262 |
| MsMI_004_Ctrimmed | 10296 | 2835724 | 2777546 | 1550457 | 10296 |
| MsMI_052_Atrimmed | 10981 | 3030948 | 2959445 | 1680340 | 10981 |
| MsMI_052_Btrimmed | 10635 | 2640338 | 2590091 | 1462320 | 10635 |
| MsMI_052_Ctrimmed | 10329 | 2697952 | 2639761 | 1399684 | 10329 |
| MsPY_031_B2trimmed | 10243 | 3397276 | 3288815 | 1965688 | 10243 |
| MsPY_031_Btrimmed | 10283 | 5201297 | 5011043 | 2547506 | 10283 |
| MsPY_031_Ctrimmed | 11330 | 2724228 | 2659996 | 1554116 | 11330 |
| MsSF_020_Atrimmed | 10228 | 2584378 | 2537296 | 1438610 | 10228 |
| MsSF_020_Btrimmed | 10514 | 2472242 | 2397755 | 1367544 | 10514 |
| MsSF_020_Ctrimmed | 10209 | 2552474 | 2505623 | 1429208 | 10209 |
| MsSF_021_A2trimmed | 9810 | 2234765 | 2187357 | 1100743 | 9810 |
| MsSF_021_Atrimmed | 10328 | 2513715 | 2466530 | 1336199 | 10328 |
| MsSF_021_Btrimmed | 9251 | 1965241 | 1934372 | 1153721 | 9251 |
| MsSF_022_Btrimmed | 9984 | 2394242 | 2358263 | 1300488 | 9984 |
| MsSF_023_A2trimmed | 9803 | 2075017 | 2035748 | 1085728 | 9803 |
| MsSF_023_A3trimmed | 10127 | 3044224 | 2993916 | 1793000 | 10127 |
| MsSF_023_Atrimmed | 9665 | 2234128 | 2215192 | 1245410 | 9665 |

**Table S5.** Number of SNPs retained after quality filtering steps.

| **Filtering steps** | **Retained SNPs** |
| --- | --- |
| Initial count after variant calling | 91,073 |
| Biallelic SNPs only | 88,313 |
| Min-max mean depth (10x - 70x) | 54,780 |
| Genotyped successfully in 80% of individuals | 38226 |
| MAF < 0.03 | 12193 |
| SNPs under linkage disequilibrium | 9266 |
| Neutral SNPs | 9198 |
| **Total SNPs used in analyses** | **9198** |

**Table S6.** Model selection results for demographic scenarios tested in fastsimcoal2. Comparison of alternative demographic models based on the Akaike Information Criterion (AIC). The models include nomig (no migration between demes), allmig (symmetric migration rates among groups), and allmig_asim (asymmetric migration rates among groups). The table shows the number of estimated parameters (K), the log-likelihood of each model (log10L), the AIC score, the difference in AIC relative to the best-supported model (ΔAIC), and the Akaike weight (ωi).

|  | **K** | **log10L** | **AIC** | **ΔAIC** | **ωi** |
| --- | --- | --- | --- | --- | --- |
| nomig | 6 | -8596.477 | 39602.23958 | 232.9121608 | 0.00 |
| allmig | 9 | -8565.388 | 39465.06945 | 95.74202492 | 0.00 |
| allmig_asim | 12 | -8543.295 | 39369.32742 | 0 | 1.00 |

**Table S7.** Results of univariate matrix regressions with randomization for genetic differentiation (F_ST_) among populations of *M. scutellaris* in relation with climatic dissimilarity (IBE_CLIM_) and six isolation by resistance (IBR) scenarios: isolation by distance (IBD); three habitat suitability models (IBR_ENM1_, IBRE_NM2_, IBR_ENM3_); habitat suitability of host plant (IBR_HOST_) and water occurrence (IBR_WATER_).

| **Variable** | ***r* ^2^** | **β** | ***t*** | ***P*** |
| --- | --- | --- | --- | --- |
| **IBD** | 0.41 | 0.83 | 16.76 | 0.001 |
| **IBE_CLIM_** | 0.61 | 0.81 | 25.26 | 0.001 |
| **IBR_ENM_** | 0.49 | 0.71 | 19.61 | 0.001 |
| **IBR_HOST_** | 0.25 | 0.55 | 11.58 | 0.001 |
| IBR_WATER_ | 0.02 | 0.01 | 2.93 | 0.176 |


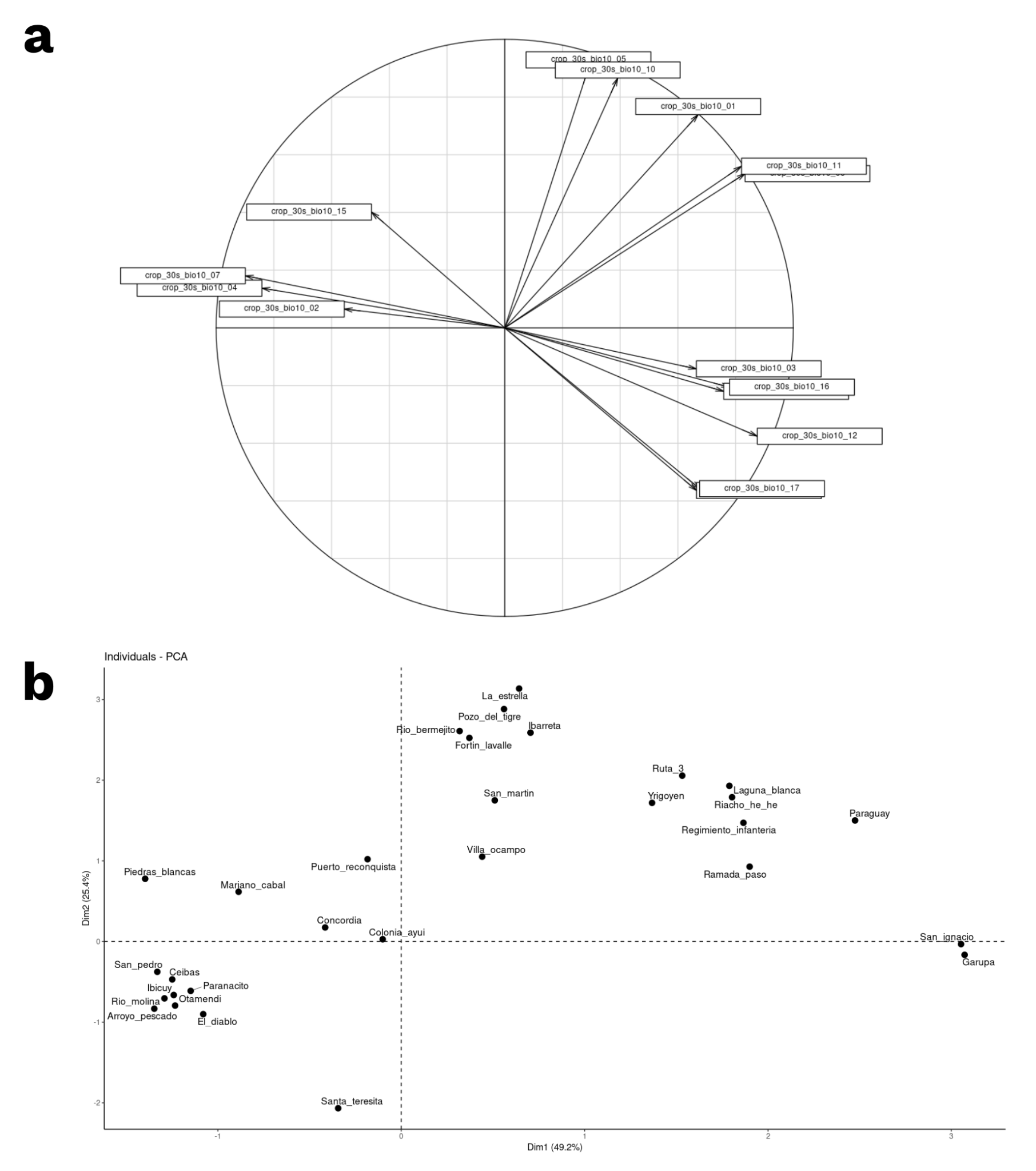


**Figure S3**. Climatic variation across sampling sites. (A) Correlation circle showing the contribution of each climatic variable to the first two principal components of the environmental PCA. (B) PCA of the 30 sampling localities of *M. scutellaris*, based on their climatic profiles. Sampling localities form distinct clusters broadly corresponding to the three main genetic lineages (Delta, Chaco, and Atlantic Forest), highlighting climatic differentiation across regions.

**Table S8.** Pearson correlation coefficients and associated p-values for each CHELSA bioclimatic variable against the first two principal components derived from a PCA including 30 sampling localities and 500 random background points. Variables were considered strong contributors to a given axis when they showed both a significant correlation (*p* < 0.05) and a correlation coefficient > 0.8.

|  | **PC1** | **p-value** | **PC2** | **p-value** |
| --- | --- | --- | --- | --- |
| **Bio1** | 0.6703 | 2.2E-16 | 0.7391 | 2.2E-16 |
| **Bio2** | -0.5561 | 2.2E-16 | 0.0652 | 0.134 |
| **Bio3** | 0.6640 | 2.2E-16 | -0.1422 | 0.001 |
| **Bio4** | -0.8413 | 2.2E-16 | 0.1374 | 0.002 |
| **Bio5** | 0.2900 | 9.9E-16 | 0.9007 | 2.2E-16 |
| **Bio6** | 0.8328 | 2.2E-16 | 0.5344 | 2.2E-16 |
| **Bio7** | -0.8989 | 2.2E-16 | 0.1804 | 2.9E-05 |
| **Bio10** | 0.3917 | 2.2E-16 | 0.8657 | 2.2E-16 |
| **Bio11** | 0.8205 | 2.2E-16 | 0.5602 | 2.2E-16 |
| **Bio12** | 0.8751 | 2.2E-16 | -0.3756 | 2.2E-16 |
| **Bio13** | 0.7588 | 2.2E-16 | -0.2200 | 3.1E-07 |
| **Bio14** | 0.6646 | 2.2E-16 | -0.5636 | 2.2E-16 |
| **Bio15** | -0.4618 | 2.2E-16 | 0.4013 | 2.2E-16 |
| **Bio16** | 0.7789 | 2.2E-16 | -0.2057 | 1.8E-06 |
| **Bio17** | 0.6752 | 2.2E-16 | -0.5573 | 2.2E-16 |
